# Genome Analysis and Physiological Characterization of Four *Streptococcus thermophilus* Strains Isolated From Chinese Traditional Fermented Milk

**DOI:** 10.3389/fmicb.2020.00184

**Published:** 2020-02-28

**Authors:** Tong Hu, Yanhua Cui, Yishuang Zhang, Xiaojun Qu, Chunyu Zhao

**Affiliations:** ^1^Department of Food Science and Engineering, School of Chemistry and Chemical Engineering, Harbin Institute of Technology, Harbin, China; ^2^Institute of Microbiology, Heilongjiang Academy of Sciences, Harbin, China

**Keywords:** *Streptococcus thermophilus*, genome comparison, technological property, antibiotic resistance, acid resistance capacity, exopolysaccharide (EPS) production, γ-aminobutyric acid (GABA) biosynthesis

## Abstract

*Streptococcus thermophilus* plays important roles in the dairy industry and is widely used as a dairy starter in the production of fermented dairy products. The genomes of *S. thermophilus* strains CS5, CS9, CS18, and CS20 from fermented milk in China were sequenced and used for biodiversity analysis. In the present study, the phylogenetic analysis of all 34 *S. thermophilus* genomes publicly available including these four strains reveals that the phylogenetic reconstruction does not match geographic distribution as strains isolated from the same continent are not even clustered on the nearby branches. The core and variable genes were also identified, which vary among strains from 0 to 202. CS9 strain contained 127 unique genes from a variety of distantly related species. It was speculated that CS9 had undergone horizontal gene transfer (HGT) during the long evolutionary process. The safety evaluation of these four strains indicated that none of them contains antibiotic resistance genes and that they are all sensitive to multiple antibiotics. In addition, the strains do not contain any pathogenic virulence factors or plasmids and thus can be considered safe. Furthermore, these strains were investigated in terms of their technological properties including milk acidification, exopolysaccharide (EPS) and γ-aminobutyric acid (GABA) production, and *in vitro* survival capacity in the gastrointestinal tract. CS9 possesses a special *eps* gene cluster containing significant traces of HGT, while the *eps* gene clusters of CS5, CS18, and CS20 are almost the same. The monosaccharide compositional analysis indicated that crude EPS-CS5, EPS-CS9, EPS-CS18, and EPS-CS20 contain similar monosaccharide compositions with different ratios. Furthermore, CS9 was one of a few GABA-producing strains that could ferment glutamate to produce GABA, which is beneficial for improving the acid tolerance of the strain. CS18 has the most potential for the production of fermented food among these four strains because of its fast growth rate, rapid acidifying capacity, and stronger acid and bile salt resistance capacity. This study focused on the genome analysis of the four new *S. thermophilus* strains to investigate the diversity of strains and provides a reference for selecting excellent strains by use of the genome data.

## Introduction

*Streptococcus thermophilus* is a necessary starter culture for yogurt manufacturing and widely used in yogurt and cheese production worldwide (Cui et al., 2016; Uriot et al., 2017). It has rapid acidifying capacity during milk fermentation and accelerates the coagulation of milk proteins. Meanwhile, this bacterium could synthesize some beneficial molecules, such as exopolysaccharide (EPS), aromatic compounds, and formic and folic acids, which contribute to many desirable production characteristics of dairy products including viscosity, texture, flavor, and water holding capacity, etc. (Hols et al., 2005; Cui et al., 2016, 2017). Furthermore, it has been confirmed that *S. thermophilus* has various health benefits such as anti-inflammatory activity, antimicrobial activity, antioxidant activity, as well as immunomodulation (Junjua et al., 2016; Cui et al., 2017; Uriot et al., 2017). These probiotic effects have a close relationship with the biologically active molecules produced by *S. thermophilus* such as EPS and bacteriocins (Hols et al., 2005; Junjua et al., 2016; Cui et al., 2017; Uriot et al., 2017).

This bacterium has been widely studied in the past few decades due to its widespread industrial application and attractive probiotic effects. In order to meet the demands of consumers for high-quality food products as well as the needs of the fermentation industry, finding new strains with outstanding processing and functional properties is a long-term and important focus for researchers. Additionally, new strains can contribute to natural diversity (Erkus et al., 2014).

In the last two decades, a number of *S. thermophilus* genomes have been published, and the whole genome information has improved the understanding of metabolic activities of this bacterium at the molecular level, including the biosynthesis of EPS and folate (Iyer et al., 2010; Cui et al., 2017; Li et al., 2018; Xiong et al., 2019), resistance to bacteriophage (Hao et al., 2018), proteolytic system (Tian et al., 2018), and carbohydrate metabolism (Prajapati et al., 2013), etc. Furthermore, the comparative genomic analysis of different *S. thermophilus* strains with various technological properties has advanced the development of knowledge about the relationship between genetic characters and phenotypic traits (Rasmussen et al., 2008; Vendramin et al., 2017).

Antibiotics started to be utilized to treat bacterial infections 60 years ago when natural strains did not contain drug resistance genes (Hughes and Datta, 1983). However, as the application of antibiotics becomes increasingly popular, resistance to antibiotics occurs during treatment. The excessive or wrong use of antibiotics coupled with the phenomenon of gene-level transfer between bacteria makes the strain gradually develop resistance in order to cope with pressure from the external environment (Polly, 1993; Levy, 1997). *S. thermophilus* is usually used as a natural food starter, thus these strains will inevitably enter the human body or animals. Once the strain carries a transferable resistance gene, it is likely to transfer the resistance gene to pathogens in the intestine, making it extremely difficult to prevent and kill them. Therefore, the analysis of strain antibiotic resistance and antibiotic resistance genes is crucially important when evaluating whether a strain can be used as a new food starter.

In order to obtain novel strains with outstanding functional/technological characteristics, 22 *S. thermophilus* strains were isolated from traditional yogurt samples, Inner Mongolia, China (Hu et al., 2018). Four strains named CS5, CS9, CS18, and CS20 with distinctive properties were selected by means of a series of evaluations in terms of their technological properties.

In this study, the whole genomes of these four strains were sequenced and used for biodiversity analysis of the *S. thermophilus* strains. The comparative analysis of genome sequences was carried out among all the *S. thermophilus* strains available in the public database. This kind of comparative phylogenetic analysis that is used for inspection of probiotic potentials has already been done for other bacterial species, such as *Lactobacillus acidipiscis*, *Lactobacillus crispatus*, *Lactobacillus casei*, and *Lactobacillus helveticus* (Ojala et al., 2014; Fontana et al., 2018, 2019; Kazou et al., 2018). Furthermore, important technological properties of the four strains were investigated including milk acidification, antibiotic resistance, EPS and γ-aminobutyric acid (GABA) production ability, and *in vitro* survival capacity in the gastrointestinal tract (GIT).

The results indicated that phylogenetic reconstruction does not match geographic distribution as strains isolated from the same continent are even not clustered on the nearby branches. It was found that the CS9 strain possessed a large number of unique genes, including a distinctive *eps* gene cluster and genes associated with replication, recombination, self-repair, etc. Therefore, CS9 could be used as a material for the research of evolution.

By screening and comparing the antibiotic resistance genes of these four strains and testing the sensitivity of the strains to 12 common antibiotics, it was found that these four strains did not contain any antibiotic resistance genes and that all of these 12 antibiotics showed a high degree of sensitivity. The results from the prediction and analysis of virulence factors and transposon indicate that the virulence factors in these four strains are mostly related to the synthesis and transport of active substances such as EPS as well as cell metabolism and thus are not pathogenic. Moreover, most of the transposase genes have been degenerated into pseudogenes due to frameshift mutations and therefore are unable to synthesize transposase which may transfer harmful genes. The remaining genes that can function are homologous to the genomes of known food-grade *S. thermophilus*. Based on the results of the safety evaluation, it can be concluded that all of these four strains of *S. thermophilus* can be considered safe; they do not cause antibiotic contamination in nature and do not contain or transport pathogenic genes. Thus, these four strains can be safely used as a natural food starter.

Furthermore, CS9 was one of the few GABA-producing strains that could ferment glutamate to produce GABA which can improve the acid tolerance of the strain. At the same time, among these four strains, CS18 has the most potential for the production of fermented food due to its fast growth rate, rapid acidifying ability, and stronger acid and bile salt resistance capacity.

## Materials and Methods

### Bacterial Strain and Growth Conditions

*Streptococcus thermophilus* strains CS5, CS9, CS18, and CS20 were isolated from yogurt in our previous study (Hu et al., 2018). They were maintained in M17 medium along with 30% (v/v) glycerol at −80°C and propagated at 37°C for three generations before use. The cultures were transferred to sterilized skimmed milk (Fine Life, MCC Trading International GmbH, Germany) for fermentation experiments.

### Genome Sequencing and Analysis

The whole genome sequencing of CS5, CS9, CS18, and CS20 was performed using a combined sequencing platform of Illumina platform and Pacbio RSII. Illumina PE library and PacBio library were constructed, respectively (Table 1). CS5 was sequenced by Illumina Miseq and Pacbio RSII. CS9, CS18, and CS20 were sequenced by Illumina Hiseq and Pacbio RSII. The whole genome map of the four strains was then completed by bioinformatics analysis after the quality control. The results were then assembled and reintegrated by Summer software. Genome annotation was performed using NCBI Prokaryotic Genome Annotation Pipeline^1^.

**TABLE 1:** The whole genome sequencing and assembly related information of *Streptococcus thermophilus* strains CS5, CS9, CS18, and CS20.

**Strain**	**Statistics of Illumina high quality data**				**Statistics of PacBio data**					**Scaffold no.**
			
	**Total reads**	**Total bases**	**Q20(%)**	**Average coverage**	**Total reads**	**Total bases**	**Largest**	**Average length**	**Average coverage**	
CS5^a^	5,364,018	1,259,565,326	98.83	710	909,931	2,913,157,082	40,955	3,201	1,567	1
CS9^b^	37,536,827	5,601,922,676	98.25	3,010	34,269	243,982,009	44,632	7,120	131	1
CS18^b^	29,230,586	4,364,084,356	98.33	2,347	23,784	230,347,981	47,315	7,026	123	1
CS20^b^	33,115,987	4,942,507,127	98.27	2,552	40,178	277,457,071	42,610	6,906	143	1

*^*a*^Sequencing service was provided by Personal Biotechnology, Co., Ltd. Shanghai, China. The Illumina data was obtained by Illumina Miseq. ^*b*^Sequencing service was provided by LC-BIO Technologies, Co., Ltd., Hanzhou, China. The Illumina data was obtained by Illumina Hiseq.*

The *ab initio* gene prediction method was used to get gene models which were identified using Glimmer3 (Delcher et al., 1999). Then all gene models were blasted against the non-redundant (NR in NCBI) database, SwissProt^2^, KEGG^3^ (Kanehisa et al., 2012), and COG^4^ (Tatusov et al., 2000). Genomic islands (GIs) located on the genome were detected by software IslandViewer (Langille and Brinkman, 2009). The family distribution, conserved domain, and model structure of glycosyltransferase (GTF) were analyzed using the carbohydrate-active enzymes database^5^, the Conserved Domain Database^6^, and the SWISS-MODEL server^7^. The virulence genes in the *S. thermophilus* strains were predicted based on the VFDB (Virulence Factors of Pathogenic Bacteria) database. The similarities of antibiotic resistance genes and transposases were blast on the NCBI database, and the pseudogenes were determined by the NCBI annotation.

The sequencing service of CS5 was provided by Personal Biotechnology, Co., Ltd., Shanghai, China. The other three strains were sequenced by LC-BIO Technologies, Co., Ltd., Hanzhou, China. The nucleotide sequences of CS5, CS9, CS18, and CS20 genomes were submitted to GenBank and assigned accession numbers CP028896, CP030927, CP030928, and CP030250.

Thirty *S. thermophilus* available genomes were downloaded from the NCBI database in July 2018 (Supplementary Table S1). The phylogenetic tree was built based on the whole genome sequences by means of Neighbor-Joining within the MEGA 6.0 software (Tamura et al., 2013).

### Antibiotic Sensitivity Analysis of Strains

Antibiotic sensitivity determination was carried out according to Sorokulova et al. (2008) with some modifications: 1 ml of a suspension of 1 × 10^8^ colony-forming units (CFUs)/ml was pipetted into 20 ml of melted solid M17 medium (55°C), then mixed and poured into a sterile dish. After the medium cooled and became solidified, wafers containing antibiotics (Bin He Microorganism Reagent, Co., Ltd.) were placed on the agar plate. Each plate was attached with three paper disks containing the same antibiotic. Then, those plates were incubated anaerobically for 48 h at an incubation temperature of 37°C before the diameters of the inhibition zones were measured and recorded. The diameter of the transparent circle around the antibiotic paper was compared to “*Performance Standards for Antimicrobial Susceptibility Testing*” (2013 edition) developed by the US Clinical and Laboratory Standards Institute to evaluate the sensitivity of bacteria to antibiotics. The antibiotics include streptomycin, ampicillin, erythromycin, clindamycin, oxacillin, rifampicin, amoxicillin, cefuroxime, tetracycline, chloramphenicol, penicillin, and vancomycin.

### Growth Characteristics

To determine the growth ability of strains, the cells were inoculated in M17 medium (1 L M17 liquid medium contains 5.0 g soya peptone, 2.5 g yeast extract, 5.0 g peptone, 0.5 g ascorbic acid, 5.0 g beef powder, 0.25 g magnesium sulfate, 5.0 g β-glycerol phosphate disodium salt, and 5.0 g lactose) and incubated anaerobically at 37°C overnight. The 200-μl volume of the suspension was then transferred to 10 ml M17 medium. The OD_600_ value and pH value were measured every hour for 8 h.

The acidifying activity of bacteria in milk was analyzed. The bacteria were incubated in M17 broth overnight and then were inoculated at 2% (v/v) to 250 ml sterile skimmed milk (Fine Life, MCC Trading International GmbH, Germany). The samples were then cultivated anaerobically at 42°C, and the pH was recorded every hour for 5 h. Each experiment was repeated at least three times.

### Acid and Bile Resistance of Strains

Resistance to acidic conditions of strains was measured according to the method of Clark et al. (1997) with some modifications. Exponential phase cells were collected from M17 cultures with an OD_600_ value of 0.4–0.6. Pelleted cells were washed twice with 10 mM sterile phosphate buffer (pH 7.0). The pH of the M17 medium in the experimental group was adjusted to 3.5 with 1M HCl, and for the M17 medium in the control group, pH 7.0 was used. Cells were resuspended in an equal volume of acidic M17 broth and incubated for 1 h at 42°C. Acid-treated cells were harvested by centrifugation at 12,000 *g* for 2 min and were then inoculated in fresh M17 broth. The ability of acid resistance was evaluated by the duration of the growth retardation period.

Then the tolerance for bile salts was tested according to previous studies with some optimization (Gilliland et al., 1984). M17 medium with 0.3% (w/v) ox bile in the experimental group and without the addition of ox bile in the control group was inoculated with exponential phase cells. The next steps were the same as the method used in the evaluation of acid resistance. Three replicate experiments were carried out to evaluate the reproducibility of each test.

### Characterization of Exopolysaccharide Production

#### Detection of the Special Genes in the *eps* Gene Cluster of CS9

In order to detect the special genes in the *eps* gene cluster of CS9, primers were designed based on the sequences of CS9 (DR994_01980-DR994_01840, Supplementary Table S2). Amplification conditions were as follows: initial denaturation at 94°C for 2 min followed by 30 cycles of 94°C for 30 s, 55°C for 30 s, and 72°C for 30–60 s. The final extension was carried out at 72°C for 2 min. PCR products were purified and sequenced.

#### Isolation of Exopolysaccharide of CS5, CS9, CS18, and CS20 Strains

*Streptococcus thermophilus* strains CS5, CS9, CS18, and CS20 were inoculated in 1 L M17 broth and cultivated at 37°C for 24 h. EPS was isolated according to the previous method with some modifications (Hu et al., 2018). In brief, the fermentation cultures were heated at 100°C for 15 min, and then the bacterial cells were removed by centrifugation (9,000 rpm, 20 min, 4°C). The supernatant was filtrated by a 0.22-μm membrane. The proteins in the supernatant were precipitated by adding 80% trichloroacetic acid (TCA) solution (w/v) in a final concentration of 4% (w/v) and were removed by centrifugation. The EPSs were precipitated by adding a three-time volume of precooled absolute ethanol. Next, the mixtures were stored at 4°C overnight. The precipitated EPS was then collected by centrifugation and dissolved in hot water (95°C). The sample was then dialyzed for 72 h at 4°C. Finally, the sample was freeze-dried before the concentrations of EPS, protein, and nucleic acid were determined. The sugar content was determined by the phenol-sulfuric acid method (Li and Shah, 2014).

#### Monosaccharide Composition Analysis of Exopolysaccharide

The 10 mg of crude EPS was mixed with 2 ml of 2M trifluoroacetic acid (TFA) and hydrolyzed at 110°C for 6 h. The sample was added with methanol and dried three times, and the dried residue was dissolved in 1 ml 0.3M sodium hydroxide for derivatization. Standard glucose, galactose, mannose, rhamnose, arabinose, ribose, xylose, fucose, galacturonic acid, glucuronic acid, glucosamine, and galactosamine were prepared as comparison.

The high-performance liquid chromatography (HPLC) analysis of 1-phenyl-3-methyl-5-pyrazolone (PMP)-derived monosaccharides was carried out on an Agilent 1100 HPLC system with a C18 HPLC column (4.6 mm i.d. × 250 mm, 5 μm, Agilent, United States). The PMP derivatives elution was performed with a mixture of 0.1M phosphate buffer (pH 6.4, A) and acetonitrile (B) in gradient elution mode with a flow rate of 1 ml/min at 30°C. Gradient elution condition was 85% A and 15% B, 0–10 min; 83% A and 17% B, 10–25 min. The UV absorbance of the effluent was set at 245 nm. The PMP derivatives were quantified by comparing their values of peak area to calibrated standard curves.

### Characterization of γ-Aminobutyric Acid Production

#### Detection of *gadB* Gene

DNA was extracted from cell culture using EZNA^®^ Bacterial DNA Kit (Omega Bio-Tek, Inc., Norcross GA, United States) according to the manual of the kit. The *gadB* gene was detected with the primers *gadF* (5′-ATGAATGAGA AGCTATTCAGAGAGATT-3′) and *gadR* (5′-TTAATGATGGA AGCCACTGC-3′). Amplification conditions were as follows: initial denaturation at 94°C for 2 min followed by 30 cycles of 94°C for 30 s, 51°C for 30 s, and 72°C for 45 s. The final extension was carried out at 72°C for 2 min. PCR products were purified and sequenced.

#### Quantitative and Qualitative Analysis of γ-Aminobutyric Acid

GABA was quantified using HPLC (UltiMate3000, Thermo Fisher, United States) equipped with a Hypersil^TM^ BDS C18 column (125 mm × 4.6 mm; Thermo Fisher, United States) according to the method of Li et al. (2008) with some modifications.

*S. thermophilus* was cultivated in M17 medium with the supplement of 1% monosodium glutamate (MSG) for 24 h at 37°C. Cell-free supernatant is the determination for the quantification of GABA. The supernatant was passed through a 0.22-μm membrane filter before HPLC analysis.

The derivatization reagent was prepared as follows: 15 mg ortho-phthalaldehyde (OPA) was first added to 5 ml methyl alcohol with the addition of 200 μl 2-mercaptoethanol, stored at 4°C, avoiding light. The derivative system was as follows: 200 μl fermentation supernatant, 200 μl derivatization reagent, and 600 μl 0.5M boric acid buffer solution (pH 10.4). The mixture was reacted for 2 min and then injected. The injection volume is 10 μl. The mobile phase A was 0.0129M sodium acetate buffer (pH 7.20). And the mobile phase B was proportioned according to 0.0129M sodium acetate buffer (pH 7.20): acetonitrile: methanol at the ratio of 1:2:1. During the elution, phase B increased linearly from 25 to 50% before 25 min and then increased linearly from 50 to 85% during 25–30 min. The temperature of the column was maintained at 25°C, and the absorbance of the detector was set to 338 nm for detection. The standard solution of GABA was prepared with sterilized water in the concentration of 10 g/l and diluted before use. The GABA derivatives were quantified by comparing their values of peak area to calibrated standard curves.

#### Effect of γ-Aminobutyric Acid Production on the Acid Resistance Ability of Strains

The assays were conducted according to Seo et al. (2015) with some modifications. The experimental strain CS9 with *gadB* gene grown overnight was diluted into fresh M17 media (pH 7.0) in 1% inoculation and then cultivated until the OD_600_ values reached 0.3–0.5 in order to ensure that the cells were in the logarithmic growth phase. These adapted cell cultures were inoculated into M17 media at pH 2.0 and pH 2.5 with or without 1% MSG. The initial cell density inoculated for acid treatment was nearly 5 × 10^6^ CFU per milliliter. The acid-treated cultures were then incubated at 37°C without shaking, and the samples were collected after being cultivated for 2 h. Aliquots were serially diluted, and triplicates were plated onto M17 agar plates. Colonies were counted after 24 h.

Survival rate was calculated as follows: [(CFU per ml at time 2 h)/(CFU per ml at time 0)] × 100%. The results presented are the averages of triplicate experiments with the standard deviations included. Strain CS20 without the *gadB* gene was used as the control.

### Extracellular pH and Intracellular pH Measurements

Extracellular pH (pH_ex_) of cultures was measured first by the pH meter (Sartorius pB10, Germany). And the fluorescent probe, 2′,7′-*bis*-(2-carboxyethyl)-5-(and-6)-carboxyflurescein, acetoxymethyl ester (BCECF AM, Beyotime, China) was used to label the prepared cells for the measurement of intracellular pH (pH_in_) (Huang et al., 2016). Cultures were incubated in the dark at 37°C for 30 min with the addition of 3 μM BCECF AM. Bacterial cells were washed three times with phosphate-buffered saline (PBS) buffer and were then resuspended in normal saline with 3 × 10^6^ cells. Fluorescence intensities of the stained cells were measured in the Multi-Mode Microplate Readers (SpectraMax iD3, United States) at the excitation wavelengths and emission of 488 and 535 nm, respectively (Hansen et al., 2016).

For the construction of the calibration curve, the stained cells were suspended in physiological saline having different pH values (2, 3, 4, 5, 6, 7, and 8) adjusted by hydrochloric acid (HCl). The ionophore nigericin was added to the final concentrations of 4 μM followed by incubation at 37°C for 10 min resulting in the equilibration of both potassium and proton ions across the cell membrane. The fluorescence intensities were measured the same as described above.

### Statistical Analysis

Statistical treatment of data was performed by means of the SPSS software (SPSS, Inc., Chicago, IL, United States). The homogeneous subsets were defined by means of Duncan. Subsets were established for α = 0.05.

## Results and Discussion

### Phylogenetic Reconstruction

**FIGURE 1:**
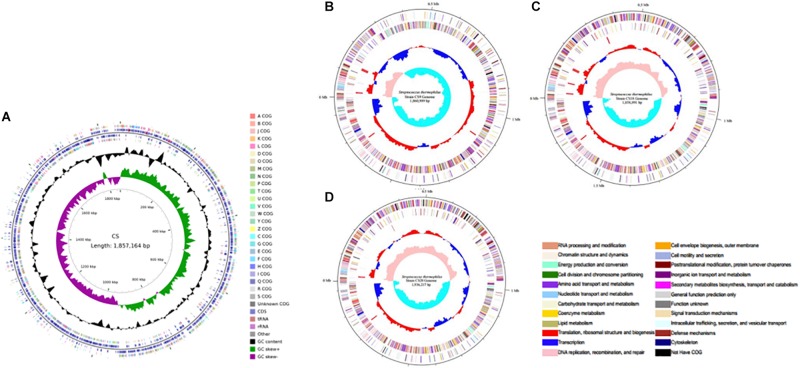
Circular genome map of the four new sequenced *Streptococcus thermophilus* chromosomes. **(A)** CS5; **(B)** CS9; **(C)** CS18; **(D)** CS20.

In this study, *S. thermophilus* strains CS5, CS9, CS18, and CS20 isolated from Chinese fermented milk were sequenced to investigate the biodiversity of the *S. thermophilus* strains (Figure 1). The genome sequencing and assembly-related information was shown in Table 1. The genome map of the *S. thermophilus* strain was completed by Illumina platform combined with Pacbio RSII. The whole genome map of the four strains was then completed by bioinformatics analysis after the quality control. The genomes of CS5, CS9, CS18, and CS20 were identified to contain 1,857,164, 1,860,988, 1,858,890, and 1,936,216 bases, respectively.

A comparative analysis of 34 *S. thermophilus* genomes available in public databases including these four new sequenced genomes was performed (Supplementary Table S1). It was found that the length of *S. thermophilus* genomes ranged from 1.73 to 2.07 Mb, while the G + C% contents were all around 39%. It is worth mentioning that CS20 is the second-largest genome of these 34 *S. thermophilus* strains just after strain M17PTZA496.

**FIGURE 2:**
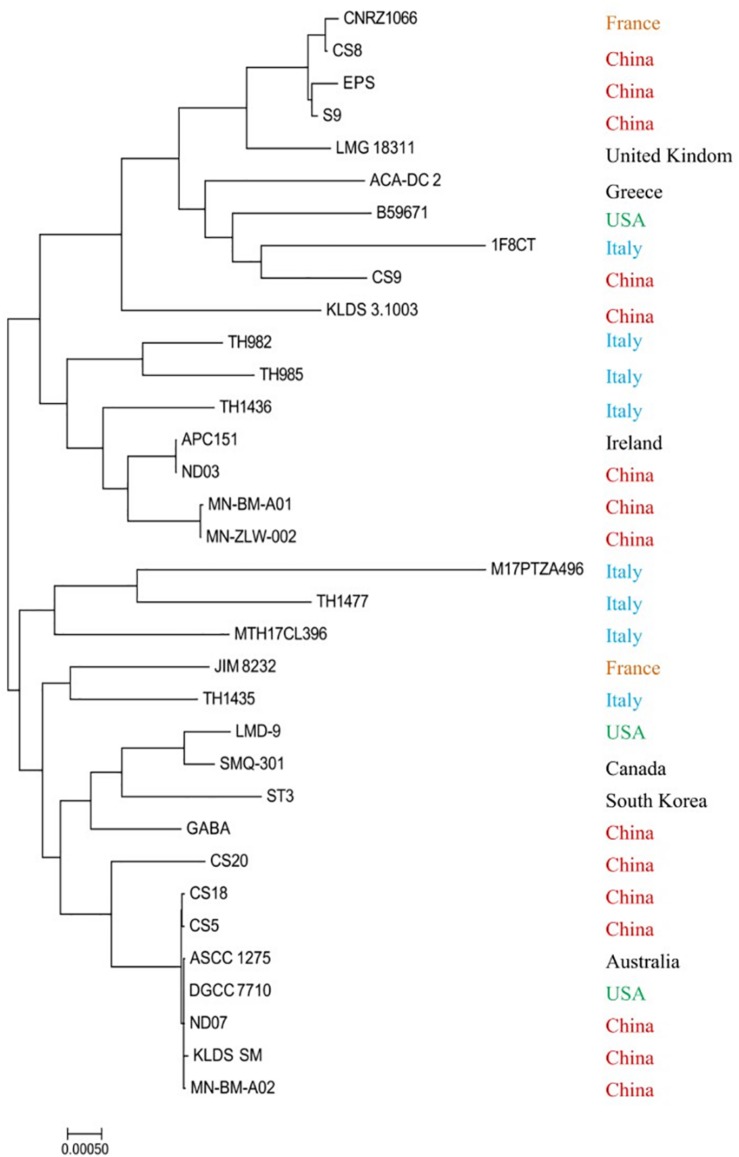
Phylogenetic trees of 34 *Streptococcus thermophilus* strains.

Most strains were isolated from fermented milk or other related milk products except the strains APC 151 and ST3, which were acquired from the digestive tract of a marine fish and commercial dietary supplements, respectively. The phylogenetic analysis was performed based on the conserved proteins detected in all tested genome sequences (Figure 2). It is clear that *S. thermophilus* strains from China distributed in various branches. They are from different provinces or areas of China; their growth environment and conditions are quite different resulting in their distinctive characters during the process of evolution. Both B59671 and LMD-9 are from the United States, but they are located so far away from each other. The similar situation also occurs in CNRZ1066 and JIM8232, which are from France. Thus, it could be concluded that the phylogenetic reconstruction based on the sequenced genomes of strains was not consistent with their geographic distribution: strains from different areas may be clustered on the same branch, while those from the same country are not necessarily located on the nearby branch. The result is consistent with that of a previous study (Vendramin et al., 2017).

In addition, CS5, CS18, and CS20 show a high proximity, while CS9 is far away from them. Specifically, CS9 is phylogenetically close to European and United States strains especially to the strain 1F8CT isolated from Italian Grana Padano Dop cheese. It reveals that the strains might not be clustered together even though they are isolated from the same region and source. It could be suggested that the long-term evolutionary process probably contributed to the difference of CS9 from the other strains.

### Identification of Core Genes and Unique Genes

**FIGURE 3:**
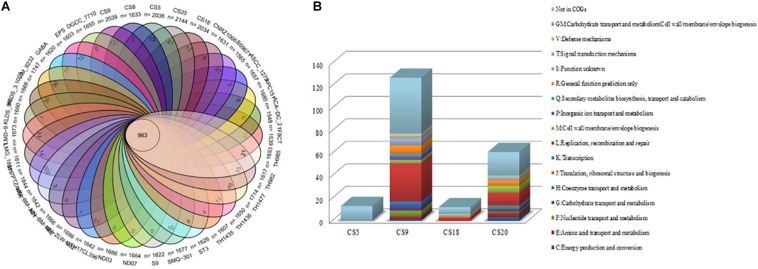
Core genes and unique genes of *Streptococcus thermophilus* strains. **(A)** Venn diagram analysis of 34 *S. thermophilus* strains. **(B)** Unique genes of four new sequenced *S. thermophilus* strains into COG categories.

Venn diagram analysis indicated that the 34 strains of *S. thermophilus* contain 963 core genes and specific genes from 0 to 202 in different strains (Figure 3A and Supplementary Table S3). Thirteen, 127, 12, and 61 unique genes were identified on CS5, CS9, CS18, and CS20 genomes, respectively. Among all tested strains, CS9 possessed the second-largest amount of unique genes, following strain M17PTZA496 harboring 202 special genes.

The unique genes of CS5, CS9, CS18, and CS20 were found to be involved in diverse functions according to the COGs annotations (Figure 3B). Most of the specific genes of CS9 belonged to the family L which is related to replication, recombination, and repair of the cells. The abundant genes associated with cell replication and modification could increase the survival ability of strains when facing the change in environmental stress during the long-term evolutionary process. The results indicated that CS9 represents the robust and most evolved among all the four strains.

Horizontal gene transfer is defined as the transmission of genetic materials between distantly related organisms in phylogenetic evolution (Goldenfeld and Woese, 2007). These unique genes were probably acquired by HGT. For instance, a heavy metal transporter CzcA (DR994_03030) predicted in CS9 genome is responsible for the transport of heavy metal ions, and it shows 100% similarity to a gene of *Streptococcus pneumoniae*. It was speculated that CzcA may be obtained from *S. pneumoniae via* HGT. HGT could play a key role in improving technological properties of *S. thermophilus* strains by acquiring new genes during the process of evolutionary and environmental adaptation (Hols et al., 2005). A lot of HGT events have been found in the *S. thermophilus* strains, which give strains new characteristics and environmental adaptability (Hols et al., 2005; Liu et al., 2009; Eng et al., 2011).

**FIGURE 4:**
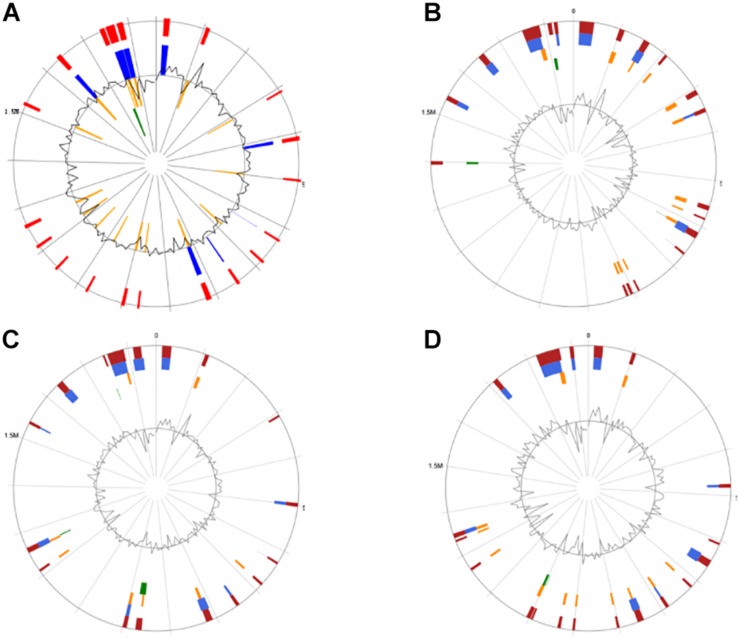
Circular map of the four *Streptococcus thermophilus* genome as generated by IslandViewer. Highlighted regions correspond to genomic islands (GIs). **(A)** CS5; **(B)** CS9; **(C)** CS18; **(D)** CS20.

Genomic islands might predict the regions of HGT in the bacterial chromosome (Juhas et al., 2009). GIs located on these four genomes were identified by the IslandViewer software (Figure 4). It was found that CS5, CS9, CS18, and CS20 contained 20, 19, 17, and 19 integrated GIs, respectively. The integrated GIs of these four strains harbored 255, 288, 286, and 306 genes, respectively.

### Safety Analysis: Virulence Factors, Antibiotic Resistance Genes, Transposable Element, and Plasmids

The safety of strains is very important for their application. In the present study, presence or absence of antibiotic resistance genes or virulence factors, mobilome (number of transposons and plasmids) has been discussed.

The sequences of the *S. thermophilus* CS5, CS9, CS18, and CS20 genomes have shown that none of the four strains contains any antibiotic-resistant genes. Although there is a tetracycline transporter (multidrug-efflux transporter) on the antibiotic gene islands of CS5 (C1A39_09480), CS18 (DTA40_09485), and CS20 (DTA54_09390), tetracycline resistance genes have not been detected. Therefore, it could be suggested that CS5, CS9, CS18, and CS20 do not contain any antibiotic resistance and that they do not cause antibiotic contamination when used as a food starter (Toomey et al., 2010).

In order to further verify the results of the analysis of the gene level, 12 common antibiotics were selected to study the resistance of these four *S. thermophilus* strains, and the results were shown in Table 2. As can be seen from the results, *S. thermophilus* CS5, CS9, CS18, and CS20 are all sensitive to these 12 common antibiotics, which is consistent with the results of the genomic analysis. This has confirmed that these four strains will not cause antibiotic contamination if used in food production.

**TABLE 2:** Antibiotic sensitivity analysis of strains.

**Strain**	**Streptomycin**	**Ampicillin**	**Erythromycin**	**Clindamycin**	**Oxacillin**	**Rifampicillin**	**Amoxicillin**	**Tetracycline**	**Chloramphenicol**	**Cefuroxime**	**Penicillin**	**Vancomycin**
CS5	26.83 ± 0.59/S	31.83 ± 0.7/2S	28.18 ± 0.26/S	29.96 ± 0.25/S	21.02 ± 0.44/S	31.71 ± 0.34/S	36.42 ± 0.28/S	22.00 ± 0.45/S	26.54 ± 1.39/S	39.01 ± 0.38/S	36.70 ± 0.28/S	21.03 ± 0.59/S
CS9	26.88 ± 0.30/S	29.01 ± 0.21/S	26.17 ± 0.77/S	23.91 ± 0.69/S	19.23 ± 0.55/S	26.99 ± 0.40/S	31.73 ± 0.34/S	25.99 ± 0.54/S	21.81 ± 0.26/S	25.98 ± 0.58/S	37.05 ± 0.59/S	24.17 ± 0.22/S
CS18	27.77 ± 0.59/S	31.19 ± 0.62/S	22.33 ± 1.27/S	22.53 ± 0.98/S	20.11 ± 1.73/S	24.84 ± 1.05/S	29.39 ± 0.27/S	21.47 ± 0.77/S	22.81 ± 0.26/S	24.17 ± 0.52/S	41.05 ± 0.59/S	22.34 ± 0.29/S
CS20	27.16 ± 0.80/S	32.33 ± 1.83/S	22.33 ± 1.27/S	21.15 ± 0.31/S	21.11 ± 1.73/S	23.69 ± 0.29/S	33.89 ± 0.12/S	21.15 ± 0.95/S	34.49 ± 1.25/S	33.17 ± 0.29/S	36.53 ± 0.25/S	20.61 ± 0.12/S

*The diameter of transparent circle around the antibiotic paper was measured (mm). Each value represents the mean of three measurements ± SD. Sensitive (S); intermediary (I); Resistance (R).*

The protein sequences of the four strains were compared against the VFDB database to obtain data on potential virulence factors on the genome (Table 3 and Supplementary Table S4). Comparison rate higher than 60% can be considered as a credible prediction. It can be seen that the number of potential virulence genes in *S. thermophilus* CS20 was the most, up to 68. The full genome length and number of genes of CS20 were the most among the four strains of *S. thermophilus*, which indicated that the genetic diversity of the CS20 genome was the most. The numbers of virulence genes in CS5, CS9, and CS18 were similar, with 48, 49, and 49 virulence genes, respectively. In general, most of these predicted potential virulence factors have a credible similarity to the *S. thermophilus* genome, with a comparison rate up to 95%. Most of these genes are associated with the synthesis and transport of EPS. *S. thermophilus* is a widely recognized food-grade microorganism; therefore, it can be inferred that the virulence genes homologous to *S. thermophilus* are not pathogenic. Studies have shown that *S. thermophilus* is different from other pathogenic streptococci, and there are generally three reasons why it is not pathogenic. Firstly, compared to other pathogenic streptococci, some of the genes in *S. thermophilus* which are involved in the sugar transport system have been degraded in the process of evolution, resulting in their inability to utilize a large number of carbohydrates (Tettelin, 2001). The ability to utilize a wide range of carbohydrates has been reported to be essential for the virulence of pathogenic streptococci, and the observed impairment of this function in *S. thermophilus* is likely to reduce its virulence potential. Also, it could be found that many streptococcal virulence-related genes (VRGs) are absent from these four *S. thermophilus* genomes or are present as only to encode basic cellular functional proteins (Bolotin et al., 2004).

**TABLE 3:** Number of genes matched with different species of bacterial in VFDB.

**Strain**	**CS5**	**CS9**	**CS18**	**CS20**
*Clostridium thermocellum*	1	1	1	1
*Chlamydia trachomatis*	1	1	1	1
*Enterococcus faecalis*	1	1	1	1
*Enterococcus faecium*	1	1	1	1
*Helicobacter acinonychis*	1	3	1	1
*Haemophilus influenzae*	–	1	–	–
*Helicobacter hepaticus*	1	–	1	1
*Haemophilus somnus*	1	–	1	1
*Listeria adhesion*	–	–	–	4
*Listeria innocua*	–	1	–	–
*Listeria ivanovii* subsp. *ivanovii*	1	–	1	–
*Listeria monocytogenes*	5	4	5	2
*Mycoplasma mycoides* subsp. *mycoides*	2	2	2	2
*Mycobacterium tuberculosis*	1	–	1	–
*Streptococcus agalactiae*	2	2	2	3
*Streptococcus mutans*	4	4	4	3
*Streptococcus pneumoniae*	2	2	2	2
*Streptococcus pyogenes*	1	2	1	1
*Streptococcus sanguinis*	–	–	–	1
*Streptococcus thermophilus*	23	24	24	42
Total	48	49	49	68

Transposases are small mobile elements that are usually found in bacterial genome and only contain their own transposition genes. Transposases are normally 0.8–2.5 kb in length and possess inverted repeats at their termini. In addition, transposases are the important factors for horizontal gene transfer (HGT) between strain genomes (Guédon et al., 1995). Thus, the vitality and similarity of transposases as well as the virulence or resistance genes around them have become important indexes for the safety evaluation.

The total numbers of transposases in *S. thermophilus* CS5, CS9, CS18, and CS20 were 132, 95, 139, and 116, respectively. But it was predicted that more than 80% of them were pseudogenes in each of them. The real numbers of transposases with a real potential function in *S. thermophilus* CS5, CS9, CS18, and CS20 were only 23, 15, 25, and 19, respectively.

Moreover, the gene blast results showed that these available transposases represent the high similarity to other established *S. thermophilus* genomes (Supplementary Table S5). As *S. thermophilus* is a recognized food-grade microorganism, these transposases are not potentially dangerous.

Plasmid is a circular DNA molecule apart from the cell chromosome as well as nuclear area DNA and is able to replicate itself in the host cell. Some plasmids could be transferred between strains, which might lead to the HGT. Therefore, the presence of plasmids in *S. thermophilus* is also an uncertain safety hazard. The detection of plasmids in the four *S. thermophilus* strains is displayed in Supplementary Figure S1. It could be seen that no plasmid was detected in these four strains. It also confirmed that all of the four strains could be safely used as natural food starters.

In summary, *S. thermophilus* CS5, CS9, CS18, and CS20 do not contain antibiotic resistance genes, virulence factors with significant pathogenicity, or plasmids that can replicate independently. In addition, these four strains contain few transposon genes. This indicates that the four strains CS5, CS9, CS18, and CS20 are not potentially toxic, and therefore they can be safely used as a novel natural food starter.

### Carbohydrate Utilization and Sugar Transport System

Exopolysaccharide biosynthetic pathways contain the transportation and phosphorylation of sugars, the formation of sugar nucleotides, the synthesis of repeating units, the polymerization of repeating units, and the exportation of EPS (Laws et al., 2001).

**FIGURE 5:**
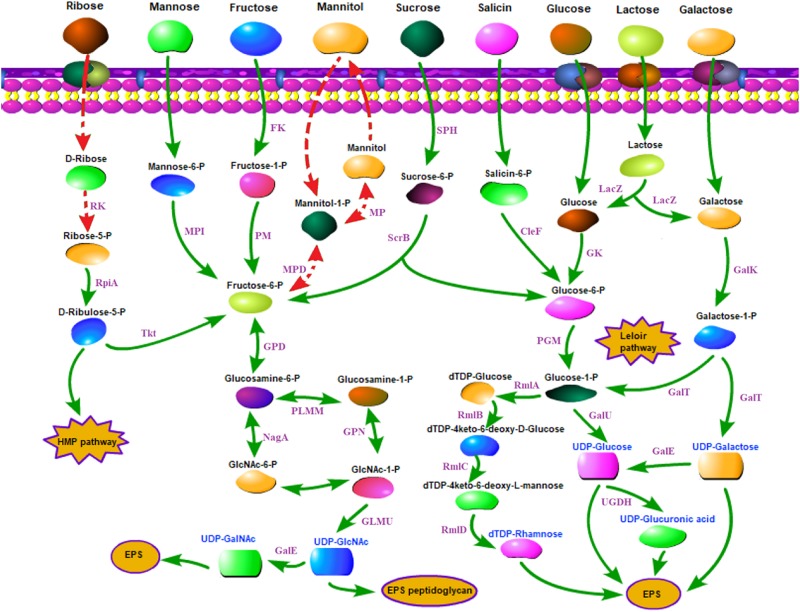
Carbohydrate utilization and sugar transport system in *Streptococcus thermophilus* strains CS5, CS9, CS18, and CS20. CelF, 6-phospho-beta-glucosidase; Fk, fructokinase; GalE, UDP-galactose-4-epimerase; GalK, galactokinase; GalT, UTP-galactose-1-phosphate uridylyltransferase; GalU, UDP-glucose pyrophosphorylase; Gk, glucokinase; GLMU, *N*-acetylglucosamine-1-phosphate uridyltransferase; GPD, glucosamine-6-phosphate deaminase; HMP pathway, pentose phosphate pathway; LacZ, beta-galactosidase; MP, mannitol-1-phosphatase; MPD, mannitol-1-phosphate 5-dehydrogenase; MPI, mannose-6 phosphate isomerase; NagA, *N*-acetyl-alpha-D-galactosaminidase; PGM, α-phosphoglucomutase; PlMM, phosphoglucosamine mutase; RK, ribokinase; RmlA, glucose-1-phosphate thymidylyltransferase; RmlB, dTDP-glucose 4, 6-dehydratase; RmlC, dTDP-4-dehydrorhamnose 3,5-epimerase; RmlD, dTDP-4-keto-L-rhamnose reductase; RpiA, ribose-5-phosphate isomerase; ScrB, sucrose6-phosphate hydrolase; SPH, sucrose-6-phosphate hydrolase; Tkt, transketolase; UDP-GalNAc, UDP-*N-*acetylgalactosamine; UDP-GlcNAc, UDP-*N-*acetylglucosamine; UGDH, UDP-glucose 6-dehydrogenase. Double-headed arrow indicates the bidirectional reaction. Arrow indicates a reaction. Red dashed arrows indicate incomplete or inactive pathways in *S. thermophilus*. Purple fonts indicate enzyme. Blue fonts indicate nucleotide sugars in *S. thermophilus*.

The carbohydrate utilization and sugar transport systems of CS5, CS9, CS18, and CS20 were analyzed (Figure 5). *S. thermophilus* takes up carbohydrates through a phosphoenolpyruvate (PEP)-phosphotransferase system (PTS) or a secondary transport system (Cui et al., 2016). The PEP-PTS consists of a PEP-dependent phosphotransferase (enzyme I, EI), a histidine-containing phosphocarrier protein (HPr, heat-stable protein), and a sugar-specific permease (transporter), which is a membrane-bound complex known as enzyme II (EII). These four genomes all harbor a PEP-dependent phosphotransferase and the HPr (Figure 5). Furthermore, genes responsible for sucrose, mannose, and fructose PTS transporter sugar-specific permease enzymes were detected in these genomes (Figure 5). Therefore, sucrose, mannose, and fructose could be transported by specific PEP-PTSs.

It was identified previously that CS5, CS18, and CS20 showed the ability to ferment a broad range of carbohydrates; they could metabolize glucose, fructose, lactose, sucrose, maltose, D (+) mannose, mannitol, D (−) salicin, melezitose, ribose, and sorbitol (Hu et al., 2018). Additionally, CS18 and CS20 could utilize D (+) galactose. CS9 can only use five kinds of sugars, including glucose, fructose, lactose, D (+) mannose, and melezitose (Hu et al., 2018).

For sugars without a specialized PEP-PTS transport system, bacteria transport them through non-PEP-PTS transport systems, i.e., primary and secondary transport systems (Laws et al., 2001). In *S. thermophilus*, sugars are mainly transported by means of permease. Genes encoding lactose/galactose permease and glucose permease were identified in these four genomes (Figure 5).

Lactose is the only carbohydrate in milk, and its content is up to 4.2% in milk, and is the pref**e**rred carbon source of *S. thermophilus* because of the long-term adaptation of strains in milk environment. Lactose is converted to glucose and galactose by β-galactosidase (LacZ) in *S. thermophilus*. The LacZ and the related transport of lactose have been identified in all the four strains in the present studies (Figure 5). *S. thermophilus* Gal-positive strains are used to reduce the browning defects even to inhibit the growth of undesirable strains (Mukherjee, 1994; de Vin et al., 2005). However, *S. thermophilus* Gal-positive strains are not common (de Vin et al., 2005; Hou et al., 2017). de Vin et al. (2005) had reported that only eight strains among the 49 tested *S. thermophilus* strains were Gal-positive. Hou et al. (2017) also found only 8% of 51 strains of *S. thermophilus* isolated from the Moorepark culture collection could utilize galactose. Therefore, the screening and identification of new Gal-positive *S. thermophilus* are desirable for food fermentation. As CS18 and CS20 could utilize galactose, they could be used in the production of cheese in order to eliminate or reduce the adverse influences of galactose accumulation.

The Leloir pathway is the most common route for galactose utilization in *S. thermophilus* (Hutkins and Morris, 1987). Galactose was converted into glucose 1-phosphate by the *galRKTEM* gene cluster and then entered the glycolysis process or other anabolic pathways (Hutkins and Morris, 1987). It is usually recognized that *gal* promoter plays an important role in the galactose catabolism (Vaughan et al., 2001).

**FIGURE 6:**
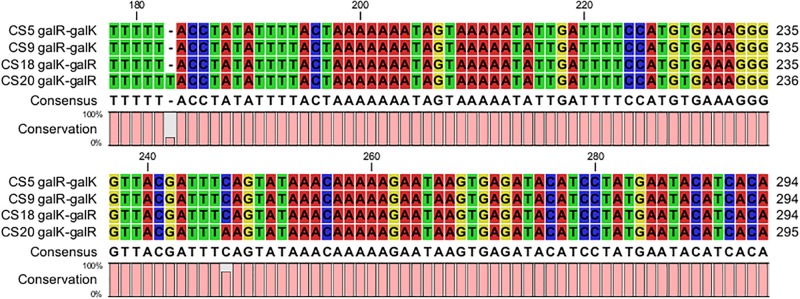
Alignment of the nucleotide sequences in the *galR–galK* intergenic region of *Streptococcus thermophilus* strains CS5, CS9, CS18, and CS20.

The DNA fragments about 437 bp containing both of the *galR* and *galK* promoter regions of CS5, CS9, CS18, and CS20 were picked out from their genome sequences. Most of these sequences in the *galR-galK* intergenic region are completely the same except for some mutations of few bases (Figure 6). The four fragments could be classified into type I and type II. CS5, CS9, and CS18 all contain the type I sequence, while CS20 belongs to type II. This result is consistent with that of our previous research (Hu et al., 2018).

Eight different types of DNA fragments types (NS1–NS8) were detected in the *galR-galK* intergenic region of 49 *S. thermophilus* (de Vin et al., 2005). In the present study, the type I nucleotide sequences are exactly the same as the NS8 type which can be found in Gal-negative strains. Type II located on the Gal-positive strain CS20, however, is beyond the types NS1–NS8. Gal-negative strains CS5 and CS9 contain type I. The result is consistent with that of a previous study (de Vin et al., 2005). However, the Gal-positive strain CS18 also belongs to type I. This indicated that although the *gal* promoter played a key role in the galactose utilization, it did not exactly determine the Gal-positive phenotype of *S. thermophilus*.

Furthermore, CS5, CS18, and CS20 can utilize salicin except CS9. Salicin is converted to salicin-6-P by the sugar-specific IIA component of the PTS system, which was detected in all the strains except CS9 (Figure 5). The ribose-positive *S. thermophilus* strains were rarely found (Erkus et al., 2014). Interestingly, CS5, CS18, and CS20 were all able to utilize ribose. Generally, D-ribose has to be phosphorylated by ribokinase before being utilized. However, the ribokinase gene was not detected in all the three genomes. It suggested that *S. thermophilus* might utilize ribose by other non-homologous pathways, and it requires further genetic characterization.

### Exopolysaccharide Biosynthesis

#### The Comparison of *eps* Gene Cluster of Strains

**FIGURE 7:**
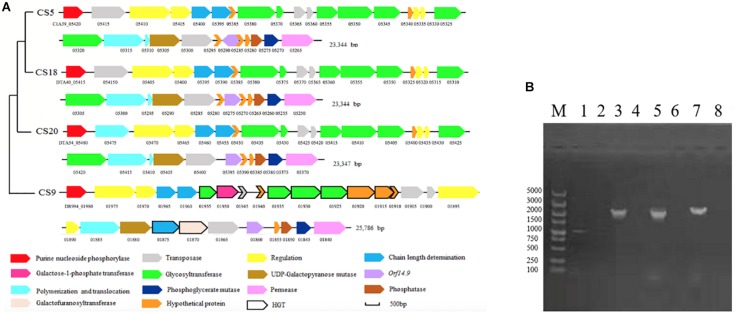
The *eps* gene cluster analysis of *Streptococcus thermophilus* strains CS5, CS9, CS18, and CS20. **(A)** Organization of *eps* gene cluster among *S. thermophilus* strains CS5, CS9, CS18, and CS20. Genes encoding the related proteins are marked with a different color. **(B)** Detection of special genes in the *eps* cluster of *S. thermophilus* CS9. M: DL5000; 1, 2: 1950-F/1950-R; 3, 4: 1920-1925-F/1920-1925-R; 5, 6:1880-1885-F/1880-1885-R; 7, 8:1930-1935-F/1930-1935-R; 1, 3, 5, 7: CS9; 2, 4, 6, 8: CS20 was used as negative control.

The *eps* gene clusters determining the EPS biosynthesis were found in CS5, CS9, CS18, and CS20 (Figure 7A). They could be divided into four parts: (1) the regulatory genes of EPS production (*epsA*, *epsB*); (2) genes for controlling the EPS chain length (*epsC*, *epsD*); (3) the EPS repeat units forming genes; (4) the genes related to polymerization and exportation of repeating units (Cui et al., 2017).

There was no significant difference among the *eps* gene clusters of CS5, CS18, and CS20 except a few bases distinctions. This result further confirmed that three *S. thermophilus* strains have close relationships. In addition, these *eps* clusters belonged to a common type and were found in some *S. thermophilus* strains, including ASCC1275, Sfi39, KLDS MS, MN-BM-A02, DGCC7710, C106, KLDS 3.1003, TH982, FI9186, and MTC310 (Cui et al., 2017). Furthermore, there was also an additional gene unit *eps2C-eps2D* in the *eps* clusters of CS5, CS18, and CS20 which could affect the length of the EPS unit.

Glycosyltransferases can take part in the transportation of various glyconucleotides. Six genes encoding GTFs were detected in the *eps* gene clusters of CS5, CS18, and CS20. They could transport UDP-glucose, UDP-galactose, dTDP-rhamnose, UDP-*N*-acetyl-glucosamine (UDP-GlcNAc), and UDP-galacofuranose to form various EPS units by glycosidic bonds.

Interestingly, the *eps* gene cluster of CS9 is unique and different from those of any known *S. thermophilus* (Figure 7A). Two regulatory EPS gene units (*eps1A-eps1B* and *eps2A-eps2B*) were found in CS9. Therefore, it was speculated that the additional *eps2A-eps2B* could improve the EPS production. To our knowledge, it has never been found in the other *S. thermophilus eps* gene clusters (Cui et al., 2017).

Especially, four GTFs of the *eps* gene cluster of CS9 have no homology to those of *S. thermophilus*. These GTFs (DR994_01925, DR994_01930, DR994_01935, and DR994_01955) originated from *Clostridium butyricum*, *Eubacteriaceae bacterium*, *Streptococcus equinus*, and *Lactococcus lactis*, respectively. DR994_01925 was predicted to belong to GTF family 2 which could transfer sugar from UDP-glucose, UDP-*N*-acetyl-galactosamine (UDP-GalNAc), or GDP-mannose to a range of substrates. DR994_01930 was a member of the GTB-type superfamily. DR994_01935 contains a conserved L-rhamnosyltransferase domain. It was speculated that EPS-CS9 might contain rhamnose. DR994_01955, however, was found similar to galactosyl transferase CpsE which could catalyze the addition of galactose to an oligosaccharide precursor or to a lipid intermediate.

At the same time, a relatively rare galactofuranose transferase (DR994_01870) was found in CS9, which was detected only in *S. thermophilus* strains 488, DSM 20617, JIM8232, and TH1436 (Delorme et al., 2011; Treu et al., 2014). It showed a high similarity to a galactofuranosyltransferase (WP_039693594.1) of *Streptococcus gallolyticus* (100% coverage, 92% identity). The results suggested that there was HGT between CS9 and *S. gallolyticus*, resulting in the exchange and integration of functional genes.

In addition, there were some other vestiges of HGT presenting in the *eps* gene cluster of CS9. For example, a gene encoding capsular biosynthesis protein (DR994_01885) was highly similar to the partial genome sequence of *Streptococcus infantarius* subsp. *infantarius* CJ18 (100% coverage, 98.11% identity); a gene encoding galactose-1-phosphate transferase (DR994_01950) showed high homology with the EPS synthesis-related gene of *L. lactis* subsp. *cremoris* NIZO B35 (100% coverage, 99.07% identity).

One IS256 transposase and four hypothetical proteins showed similarity with the dairy starter strains (*Lactobacillus delbrueckii* and *L. lactis* subsp. *lactis*), *Streptococcus macedonicus*, and *Bacillus* sp. In a word, the *eps* gene cluster of CS9 contained 12 specific genes with high homology to a variety of distantly related species, and all of them had never been detected in *S. thermophilus* strains (Supplementary Table S6). Most of these genes are located in the GIs. The GC contents of these genes are quite different from those of the CS9 genome (GC content is 38.92%). On the contrary, the GC contents of these non-*S. thermophilus* genes are similar to those of the original strains (Supplementary Table S6). It was speculated that CS9 had undergone HGT in a complex environment containing many other species during the long evolutionary process. These special genes were detected by PCR to confirm their real existence (Figure 7B). The results indicated that these specific genes predicted in CS9 genome exactly exist.

#### Monosaccharide Composition of Crude Exopolysaccharide of Strains

**FIGURE 8:**
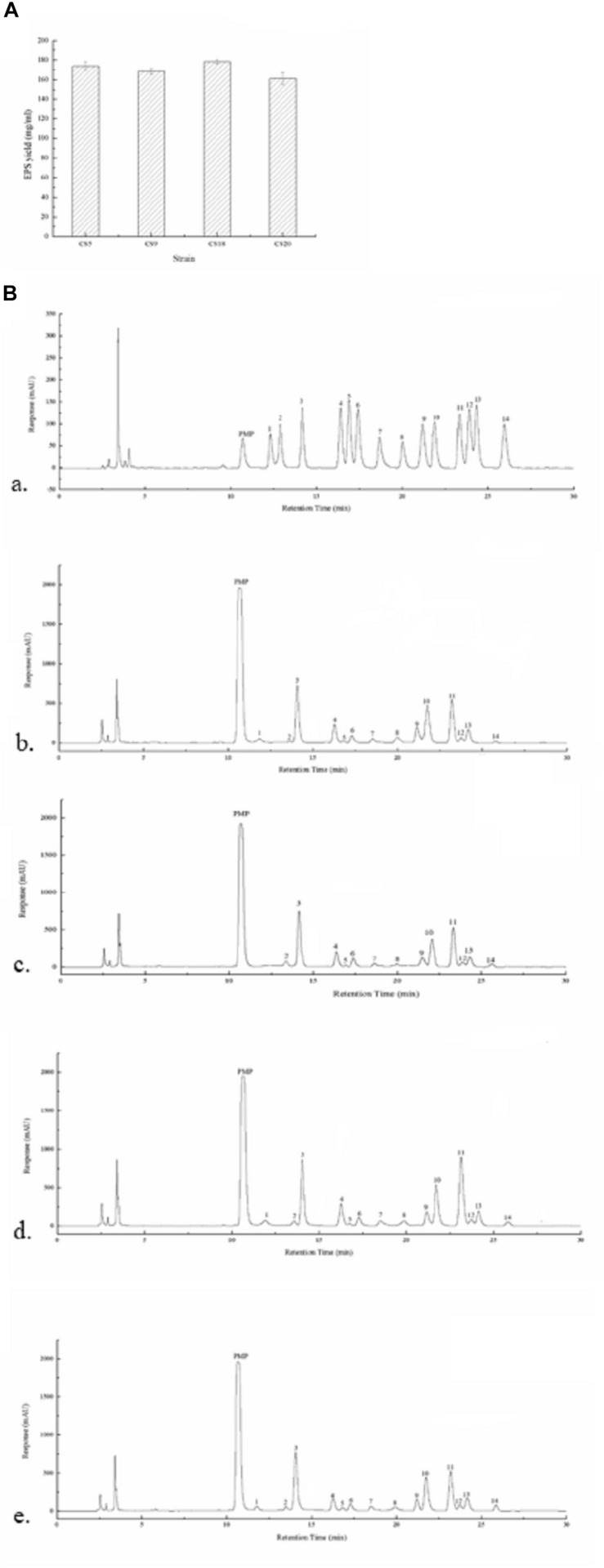
Exopolysaccharides (EPSs) produced by *Streptococcus thermophilus* strains CS5, CS9, CS18, and CS20. **(A)** The EPS yield of all the four strains. **(B)** The high-performance liquid chromatography (HPLC) chromatograms of 1-phenyl-3-methyl-5-pyrazolone (PMP) derivatives of 12 standard monosaccharides (a) and component monosaccharides released from EPS-CS5 (b); from EPS-CS9 (c); from EPS-CS18 (d); from EPS-CS20 (e). (Peaks: (1) gulouronic acid; (2) mannosuronic acid; (3) mannose; (4) ribose; (5) rhamnose; (6) glucosamine; (7) glucuronic acid; (8) galacturonic acid; (9) galactosamine; (10) glucose; (11) galactose; (12) xylose; (13) arabinose; (14) fucose.)

The EPS production ability of all the four strains in M17 medium was assayed. The crude EPS-CS5, EPS-CS9, EPS-CS18, and EPS-CS20 were 173.8, 168.3, 178.2, and 161.2 mg/ml, respectively (Figure 8A). All the four strains have similar EPS production ability in M17 medium. However, our previous studies indicated that CS9 has high EPS yield in milk (333.77 mg/ml) (Hu et al., 2018). The EPS yield of the other three strains in milk is 224.97, 213.40, and 171.95 mg/ml, respectively (Hu et al., 2018). EPS production of *S. thermophilus* is varied from 50 to 1,029 mg/L (Cui et al., 2017). Generally, more than 200 mg/L is considered high EPS production.

The monosaccharide compositions of crude EPS-CS5, EPS-CS9, EPS-CS18, and EPS-CS20 were analyzed using HPLC. Galactose, glucose, and mannose were the main monosaccharides in these crude EPSs with different ratios, and a small amount of rhamnose, ribose, xylose, fucose, galacturonic acid, glucuronic acid, glucosamine, and galactosamine were also found (Figure 8B). The results are consistent with the genetic analysis of nucleotide sugars and *eps* cluster. The *eps* gene clusters in CS5, CS18, and CS20 are almost the same, thus the monosaccharide compositions of crude EPS-CS5, EPS-CS18, and EPS-CS20 are similar. While there was no gulouronic acid detected in EPS-CS9. In detail, the composition of mannose, glucose, and galactose in EPS-CS5, EPS-CS9, EPS-CS18, and EPS-CS20 were 1.2:1:1, 0.9:0.7:1, 1.3:0.9:1, and 1.3:1:1, respectively.

The CS9 *eps* gene cluster is relatively special among these four *S. thermophilus* strains as there are some genes belonging to the non-*S. thermophilus* genomes. Usually, HGT transfers are linked to the acquisition of beneficial traits. Compared with the other three strains, the EPS production of CS9 has obvious advantage in milk. The connection between specific genes of eps gene cluster and EPS production as well as the high number of HGT events in CS9 will be further studied in the future.

### The Growth Curves and Stress Resistance of Strains

**FIGURE 9:**
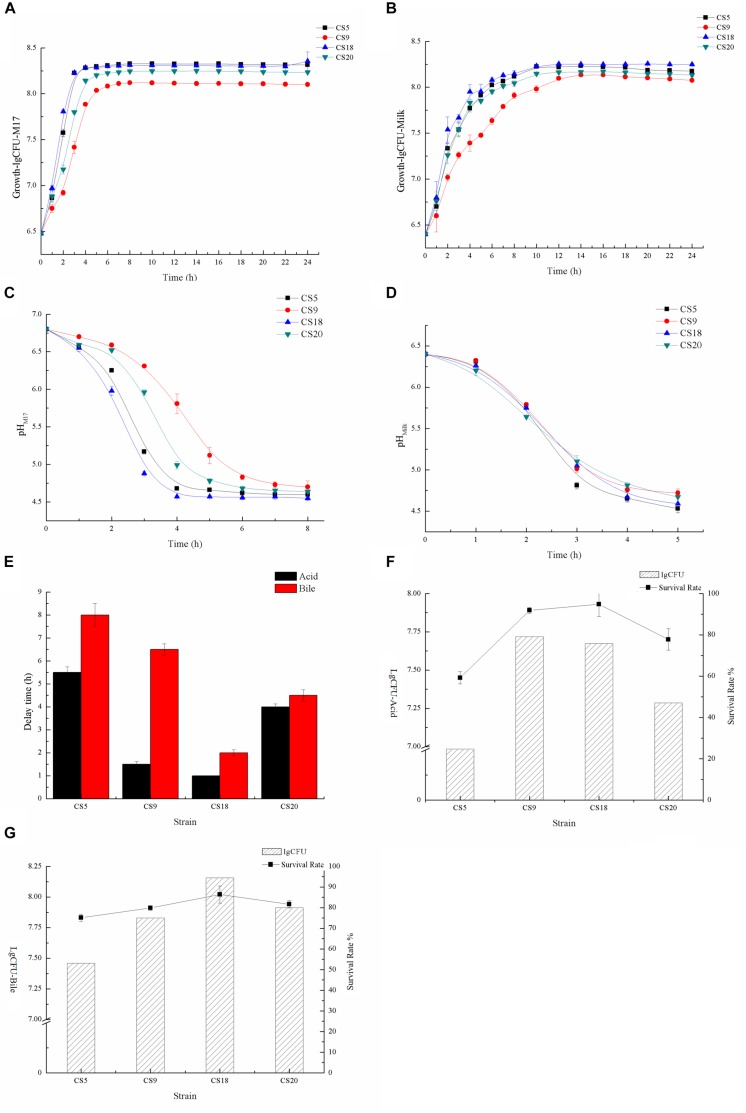
Identification of fermented characters **(A)**. Growth curves of CS5, CS9, CS18, and CS20 in M17 liquid broth **(B)**. Growth curves of CS5, CS9, CS18, and CS20 in milk **(C)**. Acidification kinetics of CS5, CS9, CS18, and CS20 in M17 liquid broth **(D)**. Acidification kinetics of CS5, CS9, CS18, and CS20 in milk **(E)**. Growth retardation period of CS5, CS9, CS18, and CS20 after stress treatment **(F)**. The viable count (lgCFU) and survival rate of CS5, CS9, CS18, and CS20 under the acid treatment **(G)**. The viable count (lgCFU) and survival rate of CS5, CS9, CS18, and CS20 under the bile treatment.

Growth dynamics of strains could reflect their technological behavior, such as the capability of milk fermentation. The growth abilities of CS5, CS9, CS18, and CS20 were measured at M17 medium (Figure 9A and Supplementary Tables S7, S8). The growth rates of CS5 and CS18 were similar. They are higher than those of CS9 and CS20 during the growth from 1 to 3 h. Among these four strains, the growth rate of CS9 was lowest. And the final biomasses of CS5 and CS18 were higher than those of CS20 and CS9 after the growth of 8 h.

Compared with the growth condition in M17, the four strains grew poorly in skimmed milk, but the growth trend of the strains was consistent with that of M17 (Figure 9B and Supplementary Tables S9, S10). The growths of *S. thermophilus* CS5, CS18, and CS20 are similar, but the growth of CS9 is obviously at a disadvantage. This might be because the key protease PrtS that could utilize casein in milk is not detected in the CS9 genome, so the growth condition of CS9 in milk was obviously weaker than the three strains CS5, CS18, and CS20 containing the *prtS* gene.

CS5 has the highest acid-producing capacity among these four strains. The pH value of the CS5 culture can reach 4.6 in less than 4 h, while the CS20 culture takes the longest time to reach pH 4.6. This result was consistent with the growth ability of strains (Figure 9C). However, the acidification rates of these four strains were similar in skimmed milk medium, and all strains reached pH 4.6 which is the attributes required for milk fermentation end point after 5 h (Figure 9D). It was speculated that milk is better for the growth of strains than M17.

The rapid acidification capability of starter culture strains was essential to guarantee the excellent characteristic of dairy products and to improve food safety by preventing the growth of pathogenic bacteria (Gaden et al., 1992). It has been known that pH 4.6 is the value required for casein coagulation and the inhibition of pathogen growth (Vendramin et al., 2017).

The growth retardation period of strain after stress treatment could reflect the acidity and bile salt resistance of the strains. Contrary to the growth situation, the tolerance of CS5 to acid and bile salt is the weakest among these four strains, and the growth retardation period were 5.5 and 8 h, respectively (Figure 9E). CS18 had the strongest adaptability to acid and bile salt stress because CS18 resumed normal growth condition only after 1 or 2 h.

It could be seen from the Supplementary Table S11 that the survival rates of these four strains were different under the same stress treatment. The acid and bile salt tolerance of the strain was measured by the value of lgCFU (Figures 9F,G and Supplementary Table S11). After the acid treatment, the number of viable bacteria of *S. thermophilus* CS18 was the highest and so was the survival rate, indicating that CS18 was the most tolerant to acid among the four strains of *S. thermophilus*. The acid tolerance of strains CS5 and CS20 was relatively low, with the survival rates of 24.67 and 47.03%, respectively. In terms of bile salt tolerance, CS18 was also the most resistant to bile salt, with a survival rate of 94.40%, while CS5 had the weakest bile salt resistance, with a survival rate as low as 53.17% after incubation with 0.3% bile salt. The results of viable counts were consistent with the results of growth lag time: the strain with a higher survival rate after stress treatment had a shorter growth delay time. The results can reflect the stress tolerance of the strains and help screen out the strains with a strong potential for colonization.

In general, CS18 had a fast growth rate, short fermentation time, and strong acid and bile salt resistance, indicating that CS18 has the potential to be used in food fermentation and can survive better in the GIT of mammals.

### Analysis of γ-Aminobutyric Acid Production

GABA represents several beneficial physiological activities in humans, and some GABA-rich products have already been used as food additives or functional food supplements (Foster and Kemp, 2006). GABA-producing *S. thermophilus* strains are uncommon in the food industry. Brasca et al. (2016) investigated 191 *S. thermophilus* strains isolated from different origins, and only a total of 20 strains (10%) harbored the *gadB* gene. Among all the strains investigated in this study apart from CS5, CS9, CS18, and CS20 strains, only seven strains (Supplementary Table S1) were found to contain *gadB* gene, including B59671, APC151, GABA, KLDS_3.1003, TH1435, ND03, and ACA-DC 2 (Sun et al., 2011; Treu et al., 2014; Alexandraki et al., 2017; Evivie et al., 2017; Linares et al., 2017; Renye et al., 2017). In these seven strains, the GABA synthesis ability of only a few strains was evaluated.

**FIGURE 10:**
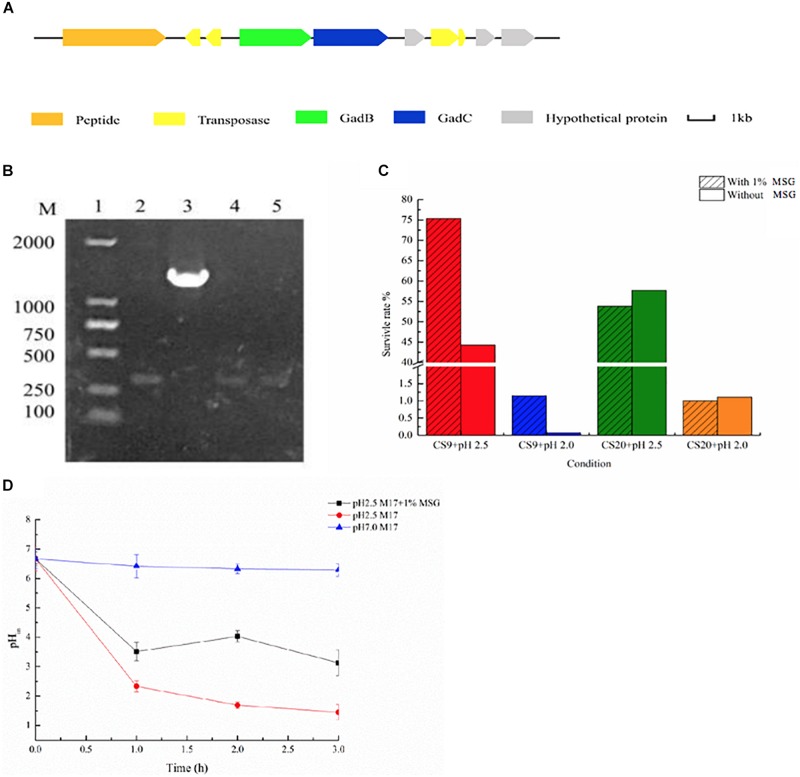
γ-Aminobutyric acid (GABA) produced by CS9. (**A**) Glutamic acid decarboxylase (GAD) system detected in CS9. (**B**) The detection of *gadB* gene of CS5, CS9, CS18, and CS20. (**C**) Effect of GABA production on the acid resistance ability of the strain (12-h cultures; acid-adapted cells) during the acid resistance assay (37°C and 2-h incubation) carried out in MRS medium (pH 2.5). Survival rate of CS9 cells under different growth conditions. (**D**) Intracellular pH (pH_in_) value of CS9 cells under different growth conditions.

GABA was synthesized by bacteria through the glutamic acid decarboxylase (GAD) system, which includes a Glu/GABA antiporter GadC, and a GAD GadA or GadB. In the present study, the genome analysis indicated that only CS9 contains a GAD system among these four strains (Figure 10A). It showed 100% similarity to those in other *S. thermophilus* genomes containing *gadB* genes (Figure 10A). In order to display the *gadB* gene in CS9 more intuitively, it was amplified (Figure 10B).

Furthermore, the GABA production ability of CS9 was analyzed. The amount of GABA produced by CS9 reached 950.36 mg/L after 24 h fermentation. It showed that CS9 could produce a competitive GABA yield comparing with other S. *thermophilus* GABA-producing strains, such as Y2 (7984.75 ± 293.33 mg/l), APC151 (2 mg/ml), and ST110 (655 μM) (Yang et al., 2008; Somkuti et al., 2012; Linares et al., 2016). Therefore, the GABA-producing *S. thermophilus* CS9 could be a new potential starter used in the production of functional foods rich in GABA by means of optimization of fermentation conditions.

Furthermore, GABA synthesis by glutamate decarboxylation in bacteria has already been found associated with acid resistance (Feehily and Karatzas, 2013; Teixeira et al., 2014). Therefore, the effect of GABA production on the acid resistance ability of strain CS9 was evaluated. The result indicated that the high-yield GABA-producing strain CS9 has a high survival rate under the serious acid treatment in the medium supplemented with MSG (Figure 10C). When cultivated in pH 2.5, the survival rate of CS9 reached 75.34% because of the production of GABA, while the rate was only 44.30% without GABA production. As for pH 2.0, CS9 could barely grow, but the survival rate of CS9 could reach 1.15% with the addition of MSG. However, there were no obvious differences in the survival rate of CS20 without *gadB* gene under the condition of pH 2.0 and pH 2.5 with the supplement of MSG.

In addition, the results showed that the pH_in_ value of CS9 cells with the supplement of 1% MSG was significantly higher than in M17 liquid medium (pH 2.5) (Figure 10D). After adding 1% MSG to the acid-treated medium, the cells consumed protons by fermenting MSG and increasing the pH_in_ value of the cells, so that the strain CS9 could adapt to the extremely acidic conditions. Furthermore, it was found that different treatment times also had an effect on the pH_in_ value of cells. The pH_in_ value of cells was the highest after 2 h incubation with 1% MSG addition. While in the first hour the strain used a few MSG to consume protons, therefore, the pH_in_ value did not change significantly. Also, the pH_in_ value decreased again after 3 h, which is probably because the overall activity of the strain was getting lower and lower when CS9 was incubated in the acidic environment for too long. The pH_in_ value of the cultures without MSG addition presented a declining trend with the accumulation of protons in cells. Obviously, CS9 could eliminate intracellular protons during the decarboxylation of glutamate by the GAD system to maintain the pH_in_ homeostasis under acidic conditions. It was speculated that CS9 could better survive in the GIT of mammals, allowing it to serve certain functions.

## Conclusion

In the present study, a comparative analysis of 34 *S. thermophilus* genomes was performed, and the influence of geographical origin on genetic variability was assessed. Results indicated that strains isolated in the same continent infrequently cluster together. Four *S. thermophilus* strains isolated from Chinese traditional fermented milk with excellent physiological and biochemical characteristics were first sequenced and analyzed. In addition, the results from the safety evaluation show that the four newly isolated *S. thermophilus* strains cannot cause antibiotic contamination in nature since they do not contain antibiotic resistance genes and are highly sensitive to many common antibiotics. Moreover, the number of virulence genes of these four strains is much fewer than that of pathogenic *Streptococcus*. There are no obvious pathogenic factors, and most of the transposon genes are gradually degenerated into pseudogenes during the evolution. The remaining transposon genes that can express normally are homologous to food-grade *S. thermophilus*, which also proves that these four strains do not have the potential to transfer harmful factors to other strains. That is to say, the safety evaluation shows that these four strains can be safely applied as a natural food starter without any adverse effects on the environment and the human body. CS9 contains a number of unique genes from the distantly related species, has a larger genome size, and represents a good candidate for the evolution research. Furthermore, CS9 can produce a high yield of GABA by the fermentation of glutamate; therefore, CS9 had a higher potential in improving human health. At the same time, it was found that CS9 could eliminate intracellular protons during the decarboxylation of glutamate by the GAD system and then maintain the pH_in_ homeostasis under acidic conditions and result in its higher survival rate. CS18 showed the fastest growth rate, the shortest fermentation time, and the strongest acid and bile salt resistance. It was suggested that CS18 has potential for food fermentation and could better survive in the GIT of mammals. At the same time, this study provides preliminary data for the study of the connection between a genotype and the phenotype (Table 4). Further research is needed in the future.

**TABLE 4:** Some genotype and the phenotype traits of the four *S. thermophilus* strains CS5, CS9, CS8, and CS20.

**Strain**	**Genome Size (Mb)**	**GC%**	**Genes**	**Growth lgCFU**		**ΔpH**		**EPS yield (mg/ml)**	** Survival Rate%**		**GABA yield (mg/L)**	***gad* gene**	**No. VRGs**	**No. transposase**	**Plasmid**	**Resistance genes**
													
				**M17 (24h)**	**Milk (24h)**	**M17 (8h)**	**Milk (5h)**		**Acid**	**Bile**						
CS5	1.86	39.08	1,978	8.314 ± 0.002	8.176 ± 0.007	2.20 ± 0.03	1.87 ± 0.04	173.80 ± 5.44	24.67	53.17	–	N	48	23	N	N
CS9	1.86	38.92	1,974	8.102 ± 0.002	8.076 ± 0.005	2.11 ± 0.04	1.68 ± 0.07	168.32 ± 3.87	79.08	75.00	950.36	Y	49	15	N	N
CS18	1.86	39.10	1,984	8.295 ± 0.003	8.248 ± 0.009	2.25 ± 0.07	1.91 ± 0.02	151.61 ± 2.89	75.81	94.40	–	N	49	25	N	N
CS20	1.94	38.90	2,043	8.233 ± 0.003	8.133 ± 0.017	2.16 ± 0.04	1.73 ± 0.08	161.22 ± 6.46	47.03	79.91	–	N	68	19	N	N

## Data Availability Statement

The datasets generated for this study can be found in the nucleotide sequences of CS5, CS9, CS18 and CS20 genomes were submitted to GenBank and assigned accession numbers CP028896, CP030927, CP030928, and CP030250.

## Author Contributions

YC conceived the idea of the study and designed the experiments. TH carried out most experiments, analyzed the data, and wrote the manuscript. YZ and CZ carried out some experiments. YC, YZ and XQ revised the manuscript.

## Conflict of Interest

The authors declare that the research was conducted in the absence of any commercial or financial relationships that could be construed as a potential conflict of interest.

## Footnotes

1 https://www.ncbi.nlm.nih.gov/genome/annotation_prok/

2 http://uniprot.org

3 http://www.genome.jp/kegg/

4 http://www.ncbi.nlm.nih.gov/COG

5 http://www.cazy.org/

6 https://www.ncbi.nlm.nih.gov/cdd/?term=

7 https://swissmodel.expasy.org/

## Supplementary Material

FIGURE S1 The detection of plasmids in the CS5, CS9, CS18, and CS20 strains. M: DL15000; 1: CS5; 2: CS9; 3: CS18; 4: CS20.

TABLE S1 The information of *Streptococcus thermophilus* strains used in this study.

TABLE S2 The primers used in the detection of the special genes in the *eps* gene cluster of CS9.

TABLE S3 Core genes and unique genes of *S. thermophilus* strains used in this study.

TABLE S4 Prediction of virulence-related genes (VRGs) in CS5, CS9, CS18, and CS20.

TABLE S5 Number of transposases in the four *S. thermophilus* strains.

TABLE S6 The analysis of specific genes in the *eps* cluster of CS9.

TABLE S7 The growth curve of the four *S. thermophilus* strains in M17^∗^.

TABLE S8 The specific growth rates and final biomasses of strains of CS5, CS9, CS18, and CS20 in M17^∗^.

TABLE S9 The growth curve of the four *S. thermophilus* strains in Milk.

TABLE S10 The specific growth rates and final biomasses of strains of CS5, CS9, CS18, and CS20 in Milk^∗^.

TABLE S11 Resistance ability of the four *S. thermophilus* strains.
