# Supplementary figures and images for: Genome Analysis and Physiological Characterization of Four Streptococcus thermophilus Strains Isolated From Chinese Traditional Fermented Milk
Source: Front Microbiol. 2020 Feb 28;11:184. doi: 10.3389/fmicb.2020.00184 (PMC7059025; doi:10.3389/fmicb.2020.00184)

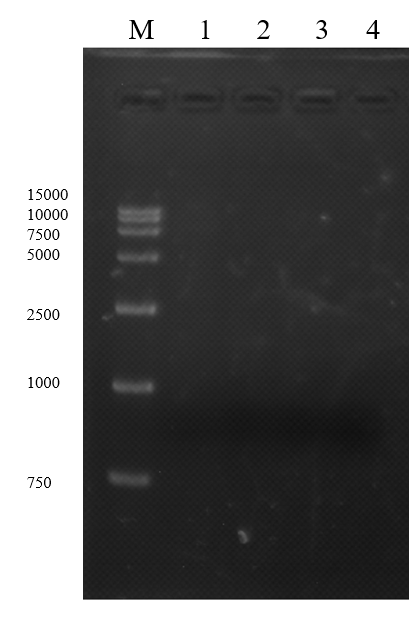

Supplement: FIGURE S1 — The detection of plasmids in the CS5, CS9, CS18, and CS20 strains. M: DL15000; 1: CS5; 2: CS9; 3: CS18; 4: CS20. [file Image_1.tiff]
