# Supplementary material for: Genome Analysis and Physiological Characterization of Four Streptococcus thermophilus Strains Isolated From Chinese Traditional Fermented Milk
Source: Front Microbiol. 2020 Feb 28;11:184. doi: 10.3389/fmicb.2020.00184 (PMC7059025; doi:10.3389/fmicb.2020.00184)
Supplement: TABLE S1 — The information of Streptococcus thermophilus strains used in this study. [file Data_Sheet_1.docx]

**Supplementary Table S1** The information of *Streptococcus thermophilus* strains used in this study.

| Strain | Genome Accession | Whole genome GC% | Genome size (Mb) | Gene number | Protein  number | Origin | Country | References |
| --- | --- | --- | --- | --- | --- | --- | --- | --- |
| CS18 | PRJNA480323 | 39.10 | 1.86 | 1,984 | 1,654 | Fermented milk | China | This study |
| CS20 | PRJNA480347 | 38.90 | 1.94 | 2,043 | 1,727 | Fermented milk | China | This study |
| CS5 | PRJNA428571 | 39.08 | 1.86 | 1,978 | 1,747 | Fermented milk | China | This study |
| CS9 | PRJNA479397 | 38.92 | 1.86 | 1,974 | 1,641 | Fermented milk | China | This study |
| 1F8CT | CM003138.1 | 39.18 | 1.75 | 1,897 | 1,542 | Cheese | Italy | Treu et al., 2014a |
| ACA DC-2 | LT604076.1 | 39.20 | 1.73 | 1,847 | 1,555 | Greek yogurt | Grace | Alexandraki et al., 2017 |
| APC 151 | CP019935.1 | 39.10 | 1.84 | 1,982 | 1,687 | Marine fish | Ireland | Linares et al., 2017 |
| ASCC 1275 | CP006819.1 | 39.10 | 1.85 | 1,974 | 1,666 | Fermented milk | Australia | Wu et al., 2014 |
| B59671 | CP022547.1 | 39.10 | 1.82 | 1,925 | 1,567 | Raw milk | USA | Renye et al., 2017 |
| CNRZ 1066 | CP000024.1 | 39.10 | 1.80 | 1,936 | 1,638 | Yogurt | France | Bolotin et al., 2004 |
| CS8 | CP016439.1 | 39.00 | 1.79 | 1,924 | 1,641 | Rubing | China |  |
| DGCC 7710 | CP025216.1 | 39.00 | 1.85 | 1,962 | 1,657 | Industrial dairy production. | USA | Hatmaker et al., 2018 |
| EPS | CP025400.1 | 39.00 | 1.81 | 1,937 | 1,608 | Milk | China |  |
| GABA | CP025399.1 | 39.10 | 1.86 | 1,952 | 1,621 | Milk | China |  |
| JIM 8232 | FR875178.1 | 38.90 | 1.93 | 2,033 | 1,748 | Milk | France | Delorme et al., 2011 |
| KLDS 3.1003 | CP016877.1 | 38.90 | 1.90 | 2,037 | 1,676 | Traditional yogurt | China | Evivie et al., 2017 |
| KLDS SM | CP016026.1 | 39.10 | 1.86 | 1,984 | 1,671 | yogurt | China | Li et al., 2017 |
| LMD 9 | CP000419.1 | 39.09 | 1.86 | 2,000 | 1,681 | Milk | America | Makarova et al., 2006 |
| LMG 18311 | CP000023.1 | 39.10 | 1.80 | 1,925 | 1,621 | Yogurt | United Kingdom | Bolotin et al., 2004 |
| M17PTZA496 | CM002372.1 | 38.87 | 2.07 | 2,274 | 1,852 | Cheese | Italy | Treu et al., 2014c |
| MN-BM-A 01 | CP012588.1 | 39.10 | 1.88 | 2,023 | 1,661 | Yogurt | China | Bai et al., 2016 |
| MN-BM-A 02 | CP010999.1 | 39.00 | 1.85 | 1,977 | 1,677 | Traditional fermented dairy product | China | Shi et al., 2015 |
| MN-ZLW-002 | CP003499.1 | 39.10 | 1.85 | 1,982 | 1,695 | Fermented Chinese dairy products | China | Kang et al., 2012 |
| MTH17CL396 | CM002371.1 | 38.99 | 1.83 | 1,963 | 1,649 | Cheese | Italy | Treu et al., 2014c |
| ND 03 | CP002340.1 | 39.00 | 1.83 | 1,968 | 1,692 | Fermented yak milk | China | Sun et al., 2011 |
| ND 07 | CP016394.1 | 39.00 | 1.87 | 1,996 | 1,684 | Fermented yak milk | China | Sun et al., 2011 |
| S9 | CP013939.1 | 39.10 | 1.79 | 1,922 | 1,630 | Traditional dairy | China |  |
| SMQ 301 | CP011217.1 | 39.10 | 1.86 | 1,993 | 1,684 | Cheese | Canada | Labrie et al., 2015 |
| ST 3 | CP017064.1 | 39.00 | 1.87 | 1,982 | 1,638 | Commercial dietary supplements | South Korea |  |
| TH1435 | CM002369.1 | 38.88 | 1.75 | 1,900 | 1,608 | Raw goat milk | Italy | Treu et al., 2014b |
| TH1436 | CM002370.1 | 38.90 | 1.78 | 1,923 | 1,650 | Raw goat milk | Italy | Treu et al., 2014b |
| TH1477 | CM003135.1 | 38.93 | 1.89 | 2,035 | 1,718 | Cheese | Italy | Treu et al., 2014a |
| TH982 | CM003136.1 | 38.89 | 1.79 | 1,917 | 1,619 | Cheese | Italy | Treu et al., 2014a |
| TH985 | CM003139.1 | 39.01 | 1.84 | 1,989 | 1,657 | Cheese | Italy | Treu et al., 2014a |

**References**

Alexandraki, V., Kazou, M., Blom, J., Pot, B., Tsakalidou, E., and Papadimitriou, K. (2017). The complete genome sequence of the yogurt isolate *Streptococcus thermophilus* ACA-DC 2. *Stand Genomic Sci.* 12, 18. doi: 10.1186/s40793-017-0227-5. eCollection 2017

Bai, Y., Sun, E., Shi, Y., Jiang, Y., Chen, Y., Liu, S., et al. (2016). Complete genome sequence of *Streptococcus thermophilus* MN-BM-A 01, a strain with high exopolysaccharides production. *J. Biotechnol*. 224, 45-46. doi: 10.1016/j.jbiotec.2016.03.003

Bolotin, A., Quinquis, B., Renault, P., Sorokin, A., Ehrlich, S. D., Kulakauskas, S., et al. (2004). Complete sequence and comparative genome analysis of the dairy bacterium *Streptococcus thermophilus*. *Nat. Biotechnol.* 22(12), 1554-1558. doi:10.1038/nbt1034

Delorme, C., Bartholini, C., Luraschi, M., Pons, N., Loux, V., Almeida, M., et al. (2011). Complete genome sequence of the pigmented *Streptococcus thermophilus* strain JIM8232. *J. Bacteriol.* 193(19), 5581-5582. doi: 10.1128/JB.05404-11.

Evivie, S. E., Li, B., Ding, X., Meng, Y., Yu, S., Du, J., et al. (2017). Complete genome sequence of *Streptococcus thermophilus* KLDS 3.1003, a strain with high antimicrobial potential against foodborne and vaginal pathogens. *Front. Microbiol*. 8, 1238. doi: 10.3389/fmicb.2017.01238

Hatmaker, E. A., Riley, L. A., O'Dell, K. B., Papanek, B., Graveley, B. R., Garrett, S. C., et al. (2018). Complete genome sequence of industrial dairy strain *Streptococcus thermophilus* DGCC 7710. *Genome Announc.* 6(6), e01587-17. doi: 10.1128/genomeA.01587-17

Kang, X., Ling, N., Sun, G., Zhou, Q., Zhang, L., and Sheng, Q. (2012). Complete genome sequence of *Streptococcus thermophilus* strain MN-ZLW-002. *J. Bacteriol.* 194(16), 4428-4429. doi: 10.1128/JB.00740-12.

Labrie, S. J., Tremblay, D. M., Plante, P. L., Wasserscheid, J., Dewar, K., Corbeil, J., et al. (2015). Complete genome sequence of *Streptococcus thermophilus* SMQ-301, a model strain for phage-host interactions. *Genome Announc.* 3(3), e00480-15. doi: 10.1128/genomeA.00480-15.

Li, B., Ding, X., Evivie, S. E., Jin, D., Meng, Y., Huo, G. et al. (2017). Short communication: genomic and phenotypic analyses of exopolysaccharides produced by, *Streptococcus thermophilus*, KLDS SM. *J. Dairy Sci.* 101(1),106-112. doi: 10.3168/jds.2017-13534

Linares, D. M., Arboleya, S., Ross, R.P., and Stanton, C. (2017). Complete genome sequence of the gamma-aminobutyric acid-producing strain *Streptococcus thermophilus* APC151. *Genome Announc,* 5(17), e00205-17. doi: 10.1128/genomeA.00205-17

Makarova, K., Slesarev, A., Wolf, Y., Sorokin, A., Mirkin, B., Koonin, E., et al. (2006). Comparative genomics of the lactic acid bacteria. *Proc. Natl. Acad. Sci.* 103(42), 15611-15616. doi: 10.1073/pnas.0607117103

Renye, J.A., Needleman, D.S., Somkuti, G. A., and Steinberg, D. H. (2017). Complete genome sequence of *Streptococcus thermophilus* strain B59671, which naturally produces the broad-spectrum bacteriocin thermophilin 110. *Genome Announc*. 5(45), e01213-17. doi: 10.1128/genomeA.01213-17

Shi, Y., Chen, Y., Li, Z., Yang, L., Chen, W., and Mu, Z. (2015). Complete genome sequence of *Streptococcus thermophilus* MN-BM-A02, a rare strain with a high acid-producing rate and low post-acidification ability. *Genome Announc.* 3(5), e00979-15. doi: 10.1128/genomeA.00979-15.

Sun, Z., Chen, X., Wang, J., Zhao, W., Shao, Y., Wu, L., et al. (2011). Complete genome sequence of *Streptococcus thermophilus* strain ND03. *J. Bacteriol.* 193(3), 793-794. doi: 10.1128/JB.01374-10

Treu, L., Vendramin, V., Bovo, B., Campanaro, S., Corich, V., Giacomini, A., 2014a. Genome sequences of four Italian *Streptococcus thermophilus* strains of dairy origin. Genome Announc. 13,2(2). pii: e00126-14. doi: 10.1128/genomeA.00126-14

Treu, L., Vendramin, V., Bovo, B., Campanaro, S., Corich, V., Giacomini, A., 2014b. Whole-genome sequences of *Streptococcus thermophilus* strains TH1435 and TH1436, isolated from raw goat milk. Genome Announc. 16;2(1). pii: e01129-13. doi: 10.1128/genomeA.01129-13

Treu, L., Vendramin, V., Bovo, B., Campanaro, S., Corich, V., Giacomini, A., 2014c. Genome sequences of *Streptococcus thermophilus* strains MTH17CL396 and M17PTZA496 from Fontina, an Italian PDO Cheese. Genome Announc. 13, 2(1). pii: e00067-14. doi: 10.1128/genomeA.00067-14.

Wu, Q., Tun, H. M., Leung, F. C., and Shah, N. P. (2014). Genomic insights into high exopolysaccharide-producing dairy starter bacterium *Streptococcus thermophilus* ASCC 1275. *Sci. Rep*. 4:4974. doi: 10.1038/srep04974

**Supplementary Table S2** The primers used in the detection of the special genes in the *eps* gene cluster of CS9

| Locus | Primer | Sequence (5’-3’) | Size (bp) |
| --- | --- | --- | --- |
| DR994_01950 | 1950-F | CAATTGAAATTAGAGATGATAC | 1063 |
|  | 1950-R | TGGTCTTGGACCAACTAATGC |  |
| DR994_01920-01925 | 1920-1925-F | TTACTTGCAATTCCTAGTGTC | 2059 |
|  | 1920-1925-R | CTCTACTTGAGTGTTTACAC |  |
| DR994_01880-01885 | 1880-1885-F | CGAATACCTTTACCGACACGC | 1981 |
|  | 1880-1885-R | CTTTGGGTAGATGGAAGTGCC |  |
| DR994_01930-01935 | 1930-1935-F | ACAGCTCTTTAGAAATAATTC | 2184 |
|  | 1930-1935-R | GCTATATGCCTAATTTTATGA |  |

**Supplementary Table S3** Core genes and unique genes of *S. thermophilus* strains used in this study

| Strain | Core genes | Special genes | Total genes |
| --- | --- | --- | --- |
| M17PTZA496 | 963 | 202 | 1844 |
| CS9 | 963 | 127 | 2039 |
| CS20 | 963 | 61 | 2144 |
| MTH17CL396 | 963 | 51 | 1642 |
| TH1477 | 963 | 48 | 1714 |
| JIM 8232 | 963 | 30 | 1747 |
| KLDS 3.1003 | 963 | 30 | 1666 |
| B59671 | 963 | 21 | 1565 |
| TH985 | 963 | 21 | 1653 |
| GABA | 963 | 17 | 1620 |
| TH982 | 963 | 15 | 1617 |
| LMG 18311 | 963 | 14 | 1611 |
| 1F8CT | 963 | 13 | 1539 |
| CS5 | 963 | 13 | 2036 |
| CS18 | 963 | 12 | 2034 |
| EPS | 963 | 12 | 1603 |
| LMD-9 | 963 | 11 | 1673 |
| TH1436 | 963 | 11 | 1650 |
| SMQ 301 | 963 | 10 | 1677 |
| ST3 | 963 | 9 | 1626 |
| ACA DC 2 | 963 | 6 | 1548 |
| TH1435 | 963 | 4 | 1607 |
| MN BM A01 | 963 | 3 | 1642 |
| ASCC 1275 | 963 | 2 | 1657 |
| KLDS SM | 963 | 1 | 1660 |
| MN ZLW 002 | 963 | 1 | 1686 |
| APC 151 | 963 | 0 | 1680 |
| CNRZ 1066 | 963 | 0 | 1631 |
| CS8 | 963 | 0 | 1633 |
| DGCC 7710 | 963 | 0 | 1655 |
| MN BM A02 | 963 | 0 | 1666 |
| ND03 | 963 | 0 | 1686 |
| ND07 | 963 | 0 | 1664 |
| S9 | 963 | 0 | 1622 |

**Supplementary Table S4** Prediction of virulence-related genes (VRGs) in CS5, CS9, CS18, and CS20

| Prediction of virulence-related genes (VRGs) in CS5 | | | | |
| --- | --- | --- | --- | --- |
| Protein | **Identity** | **Annotation** | **Similarity** | **Pseudogene** |
| C1A39_01145 | 71% | (*groEL*) chaperonin GroEL | *Clostridium thermocellum* | N |
| C1A39_00705 | 64% | (*CT396*) molecular chaperone DnaK | *Chlamydia trachomatis* | N |
| C1A39_05330 | 63% | (*cpsI*) UDP-galactopyranose mutase | *Enterococcus faecalis* | N |
| C1A39_01115 | 63% | (*uppS*) undecaprenyl diphosphate synthase | *Enterococcus faecium* | N |
| C1A39_01515 | 63% | (*ureA*) urease alpha subunit | *Helicobacter acinonychis* | N |
| C1A39_01535 | 64% | (*ureG*) urease/hydrogenase-associated predicted GTPase UreG | *Helicobacter hepaticus* | N |
| C1A39_05665 | 61% | (*galE*) UDP-glucose 4-epimerase | *Haemophilus somnus* | N |
| C1A39_09165 | 66% | (*lap*) putative alcohol-acetaldehyde dehydrogenase | *Listeria ivanovii subsp. ivanovii* | N |
| C1A39_01665 | 61% | (*lisR*) two-component response regulator | *Listeria monocytogenes* | N |
| C1A39_01855 | 64% | (*clpP*) ATP-dependent Clp protease proteolytic subunit | *Listeria monocytogenes* | N |
| C1A39_09150 | 75% | (*lap*) bifunctional acetaldehyde-CoA/alcohol dehydrogenase | *Listeria monocytogenes* | N |
| C1A39_09155 | 69% | (*lap*) bifunctional aldehyde-alcohol dehydrogenase | *Listeria monocytogenes* | N |
| C1A39_09160 | 71% | (*lap*) bifunctional aldehyde-alcohol dehydrogenase | *Listeria monocytogenes* | N |
| C1A39_02495 | 73% | (*tuf*) translation elongation factor Tu [EF-Tu (CVF587)] | *Mycoplasma mycoides subsp. mycoides* | N |
| C1A39_07095 | 64% | (*oppF*) oligopeptide ABC transporter, permease component | *Mycoplasma mycoides subsp. mycoides* | N |
| C1A39_07235 | 60% | (*sigA/rpoV*) RNA polymerase sigma factor | *Mycobacterium tuberculosis* | N |
| C1A39_01410 | 64% | (*scpB*) segregation and condensation protein B | *Streptococcus agalactiae* | N |
| C1A39_04485 | 79% | (*cpsY*) transcriptional regulator CpsY | *Streptococcus agalactiae* | N |
| C1A39_00990 | 71% | (*rgpG*) putative glycosyl transferase N-acetylglucosaminyltransferase), RgpG | *Streptococcus mutans* | N |
| C1A39_02270 | 65% | (*gbpB*) putative peptidoglycan hydrolase | *Streptococcus mutans* | N |
| C1A39_08910 | 88% | (*SMU.322c*) glucose-1-phosphate uridylyltransferase | *Streptococcus mutans* | N |
| C1A39_03315 | 95% | (*eno*) phosphopyruvate hydratase [Streptococcal enolase (CVF153)] | *Streptococcus pneumoniae* | N |
| C1A39_08665 | 95% | (*plr/gapA*) glyceraldehyde-3-phosphate dehydrogenase, type I | *Streptococcus pneumoniae* | N |
| C1A39_03750 | 65% | (*mf2*) MF2 | *Streptococcus pyogenes* | Y |
| C1A39_09900 | 63% | (*htrA/degP*) putative serine protease | *Streptococcus pyogenes* | N |
| C1A39_05385 | 79% | (*SSU98_0573*) Cps2F [Capsule (CVF186)] | *Streptococcus suis* | Y |
| C1A39_00265 | 100% | (*cbpD*) Surface antigen | *Streptococcus thermophilus* | N |
| C1A39_00860 | 100% | (*tig/ropA*) FKBP-type peptidyl-prolyl cis-trans isomerase (trigger factor) | *Streptococcus thermophilus* | N |
| C1A39_02360 | 97% | (*cbpD*) cell wall protein precursor, similar to choline binding protein | *Streptococcus thermophilus* | N |
| C1A39_02365 | 97% | (*cbpD*) cell wall protein precursor, similar to choline binding protein | *Streptococcus thermophilus* | N |
| C1A39_02775 | 99% | (*cshA*) Uncharacterized conserved phage related protein | *Streptococcus thermophilus* | N |
| C1A39_04795 | 100% | (*fbp54*) Fibronectin-binding protein | *Streptococcus thermophilus* | N |
| C1A39_05345 | 97% | (*STER_1057*) Polysaccharide Transporter, PST family | *Streptococcus thermophilus* | N |
| C1A39_05390 | 98% | (*epsE*) exopolysaccharide biosynthesis protein, glycosyl-1-phosphate transferase | *Streptococcus thermophilus* | N |
| C1A39_05400 | 98% | (*STER_1068*) Tyrosine-protein kinase (capsular polysaccharide biosynthesis) | *Streptococcus thermophilus* | N |
| C1A39_05410 | 99% | (*STER_1070*) Capsular polysaccharide biosynthesis protein | *Streptococcus thermophilus* | N |
| C1A39_05415 | 100% | (*STER_1071*) Transcriptional regulator | *Streptococcus thermophilus* | N |
| C1A39_05795 | 97% | (*cshA*) surface-associated protein cshA precursor, truncated | *Streptococcus thermophilus* | Y |
| C1A39_06100 | 100% | (*STER_1222*) dTDP-D-glucose 4,6-dehydratase | *Streptococcus thermophilus* | N |
| C1A39_06105 | 100% | (*STER_1223*) dTDP-4-dehydrorhamnose 3,5-epimerase or related enzyme | *Streptococcus thermophilus* | N |
| C1A39_06110 | 100% | (*rmlA*) glucose-1-phosphate thymidyl transferase | *Streptococcus thermophilus* | N |
| C1A39_06275 | 97% | (*srtA*) Sortase (surface protein transpeptidase) | *Streptococcus thermophilus* | N |
| C1A39_07160 | 99% | (*rgpF*) polysaccharide biosynthesis protein | *Streptococcus thermophilus* | N |
| C1A39_07165 | 93% | (*rgpE*) polysaccharide biosynthesis protein/putative glycosyltransferase | *Streptococcus thermophilus* | N |
| C1A39_07170 | 100% | (*rgpD*) polysaccharide ABC exporter ATP-binding protein | *Streptococcus thermophilus* | N |
| C1A39_07175 | 100% | (*rgpC*) polysaccharide ABC exporter membrane-spanning protein | *Streptococcus thermophilus* | N |
| C1A39_07180 | 99% | (*rgpB*) polysaccharide biosynthesis protein/putative rhamnosyl transferase | *Streptococcus thermophilus* | N |
| C1A39_07185 | 99% | (*rgpA*) polysaccharide biosynthesis protein/putative rhamnosyl transferase | *Streptococcus thermophilus* | N |
| C1A39_07200 | 63% | (*rgpX2*) polysaccharide biosynthesis protein, putative rhamnosyl transferase | *Streptococcus thermophilus* | N |
| C1A39_07205 | 94% | (*rgpX3*) polysaccharide biosynthesis protein, putative transporter | *Streptococcus thermophilus* | N |
| C1A39_07220 | 100% | (*rmlD*) dTDP-4-keto-L-rhamnose reductase | *Streptococcus thermophilus* | N |

**Prediction of virulence-related genes (VRGs) in CS9.**

| Protein | Identity | Annotation | Similarity | Pseudogene |
| --- | --- | --- | --- | --- |
| DR994_06620 | 71% | (*groEL*) chaperonin GroEL | *Clostridium thermocellum* | N |
| DR994_00685 | 64% | (*CT396*) molecular chaperone DnaK | *Chlamydia trachomatis* | N |
| DR994_01890 | 62% | (*cpsI*) UDP-galactopyranose mutase | *Enterococcus faecalis* | N |
| DR994_06655 | 63% | (*uppS*) undecaprenyl diphosphate synthase | *Enterococcus faecium* | N |
| DR994_02225 | 62% | (*galE*) UDP-glucose 4-epimerase | *Haemophilus influenzae* | N |
| DR994_06245 | 64% | (*ureG*) urease/hydrogenase-associated predicted GTPase UreG | *H.acinonychis* | N |
| DR994_06265 | 63% | (*ureA*) urease alpha subunit | *H.acinonychis* | N |
| DR994_09140 | 70% | (*lap*) hypothetical protein | *Listeria innocua* | N |
| DR994_05955 | 64% | (*clpP*) ATP-dependent Clp protease proteolytic subunit | *Listeria monocytogenes* | N |
| DR994_06140 | 61% | (*lisR*) two-component response regulator | *Listeria monocytogenes* | N |
| DR994_09135 | 73% | (*lap*) bifunctional acetaldehyde-CoA/alcohol dehydrogenase | *Listeria monocytogenes* | N |
| DR994_09150 | 71% | (*lap*) bifunctional aldehyde-alcohol dehydrogenase | *Listeria monocytogenes* | N |
| DR994_05815 | 73% | (*tuf*) translation elongation factor Tu | *Mycoplasma mycoides subsp. mycoides* | N |
| DR994_07115 | 65% | (*oppF*) oligopeptide ABC transporter, permease component | *Mycoplasma mycoides subsp. mycoides* | N |
| DR994_01205 | 61% | (*cylG*) 3-ketoacyl-ACP-reductase CylG | *Streptococcus agalactiae* | Y |
| DR994_04410 | 97% | (*ssp-5*) agglutinin receptor | *Streptococcus agalactiae* | N |
| DR994_06355 | 64% | (*scpB*) segregation and condensation protein B | *Streptococcus agalactiae* | N |
| DR994_02360 | 61% | (*cshB*) surface-associated protein CshB | *Streptococcus gordonii* | Y |
| DR994_00195 | 68% | (*gbpB*) putative peptidoglycan hydrolase | *Streptococcus mutans* | N |
| DR994_05530 | 67% | (*gbpB*) putative peptidoglycan hydrolase | *Streptococcus mutans* | N |
| DR994_06785 | 71% | (*rgpG*) putative glycosyl transferase N-acetylglucosaminyltransferase), RgpG | *Streptococcus mutans* | N |
| DR994_08905 | 88% | (*SMU.322c*) glucose-1-phosphate uridylyltransferase | *Streptococcus mutans* | N |
| DR994_03475 | 62% | (*zmpB*) zinc metalloprotease ZmpB, putative | *Streptococcus pneumoniae* | N |
| DR994_04590 | 95% | (*eno*) phosphopyruvate hydratase | *Streptococcus pneumoniae* | N |
| DR994_08670 | 95% | (*plr/gapA*) glyceraldehyde-3-phosphate dehydrogenase, type I | *Streptococcus pneumoniae* | N |
| DR994_03930 | 62% | (*mf2*) deoxyribonuclease, phage associated | *Streptococcus pyogenes* | N |
| DR994_09865 | 62% | (*htrA/degP*) putative serine protease | *Streptococcus pyogenes* | N |
| DR994_00265 | 100% | (*cbpD*) cell wall protein precursor, similar to choline binding protein | *Streptococcus thermophilus* | N |
| DR994_01040 | 79% | (*cpsY*) transcriptional regulator CpsY | *Streptococcus thermophilus* | N |
| DR994_01355 | 100% | (*fbp54*) fibronectin-binding protein-like protein A | *Streptococcus thermophilus* | N |
| DR994_01900 | 99% | (*epsB*) exopolysaccharide biosynthesis protein | *Streptococcus thermophilus* | Y |
| DR994_01905 | 97% | (*epsA*) transcriptional activator-exopolysaccharide biosynthesis | *Streptococcus thermophilus* | N |
| DR994_01960 | 98% | (*STER_1068*) Tyrosine-protein kinase (capsular polysaccharide biosynthesis) | *Streptococcus thermophilus* | N |
| DR994_01965 | 95% | (*epsC*) exopolysaccharide exporter accessory protein | *Streptococcus thermophilus* | N |
| DR994_01970 | 100% | (*epsB*) exopolysaccharide biosynthesis protein | *Streptococcus thermophilus* | N |
| DR994_01975 | 97% | (*epsA*) transcriptional activator-exopolysaccharide biosynthesis | *Streptococcus thermophilus* | N |
| DR994_02350 | 98% | (*cshA*) surface-associated protein cshA precursor, truncated | *Streptococcus thermophilus* | N |
| DR994_02355 | 96% | (*cshA*) surface-associated protein cshA precursor, truncated | *Streptococcus thermophilus* | Y |
| DR994_02585 | 100% | (*STER_1222*) dTDP-D-glucose 4,6-dehydratase | *Streptococcus thermophilus* | N |
| DR994_02590 | 100% | (*STER_1223*) dTDP-4-dehydrorhamnose 3,5-epimerase or related enzyme | *Streptococcus thermophilus* | N |
| DR994_02595 | 100% | (*rmlA*) glucose-1-phosphate thymidyl transferase | *Streptococcus thermophilus* | N |
| DR994_02755 | 96% | (*srtA*) Sortase (surface protein transpeptidase) | *Streptococcus thermophilus* | N |
| DR994_05445 | 98% | (*cbpD*) cell wall protein precursor, similar to choline binding protein | *Streptococcus thermophilus* | Y |
| DR994_05450 | 93% | (*cbpD*) cell wall protein precursor, similar to choline binding protein | *Streptococcus thermophilus* | N |
| DR994_06915 | 100% | (*tig/ropA*) trigger factor | *Streptococcus thermophilus* | N |
| DR994_07190 | 99% | (*rgpF*) polysaccharide biosynthesis protein | *Streptococcus thermophilus* | N |
| DR994_07195 | 100% | (*rgpE*) polysaccharide biosynthesis protein/putative glycosyltransferase | *Streptococcus thermophilus* | N |
| DR994_07200 | 100% | (*rgpD*) polysaccharide ABC exporter ATP-binding protein | *Streptococcus thermophilus* | N |
| DR994_07205 | 100% | (*rgpC*) polysaccharide ABC exporter membrane-spanning protein | *Streptococcus thermophilus* | N |
| DR994_07210 | 100% | (*rgpB*) polysaccharide biosynthesis protein/putative rhamnosyl transferase | *Streptococcus thermophilus* | N |
| DR994_07215 | 100% | (*rgpA*) polysaccharide biosynthesis protein/putative rhamnosyl transferase | *Streptococcus thermophilus* | N |
| DR994_07220 | 99% | (*rgpX2*) polysaccharide biosynthesis protein, putative rhamnosyl transferase | *Streptococcus thermophilus* | N |
| DR994_07225 | 98% | (*rgpX3*) polysaccharide biosynthesis protein, putative transporter | *Streptococcus thermophilus* | N |
| DR994_07240 | 100% | (*rmlD*) dTDP-4-keto-L-rhamnose reductase | *Streptococcus thermophilus* | N |

**Prediction of virulence-related genes (VRGs) in CS18**

| Protein | Identity | Annotation | Similarity | Pseudogene |
| --- | --- | --- | --- | --- |
| DTA40_01140 | 71% | (*groEL*) chaperonin GroEL | *Clostridium thermocellum* | N |
| DTA40_00705 | 64% | (*CT396*) molecular chaperone DnaK | *Chlamydia trachomatis* | N |
| DTA40_05325 | 63% | (*cpsI*) UDP-galactopyranose mutase | *Enterococcus faecalis* | N |
| DTA40_01110 | 63% | (*uppS*) undecaprenyl diphosphate synthase | *Enterococcus faecium* | N |
| DTA40_01510 | 63% | (*ureA*) urease alpha subunit | *Helicobacter acinonychis* | N |
| DTA40_01530 | 64% | (*ureG*) urease/hydrogenase-associated predicted GTPase UreG | *Helicobacter hepaticus* | N |
| DTA40_05660 | 61% | (*galE*) UDP-glucose 4-epimerase | *Haemophilus somnus* | N |
| DTA40_09175 | 66% | (*lap*) putative alcohol-acetaldehyde dehydrogenase | *L. ivanovii subsp. ivanovii* | N |
| DTA40_01660 | 61% | (*lisR*) two-component response regulator | *Listeria monocytogenes* | N |
| DTA40_01850 | 64% | (*clpP*) ATP-dependent Clp protease proteolytic subunit | *Listeria monocytogenes* | N |
| DTA40_09155 | 75% | (*lap*) bifunctional acetaldehyde-CoA/alcohol dehydrogenase | *Listeria monocytogenes* | N |
| DTA40_09160 | 69% | (*lap*) bifunctional aldehyde-alcohol dehydrogenase | *Listeria monocytogenes* | N |
| DTA40_09170 | 71% | (*lap*) bifunctional aldehyde-alcohol dehydrogenase | *Listeria monocytogenes* | N |
| DTA40_02490 | 73% | (*tuf*) translation elongation factor Tu [EF-Tu (CVF587)] | *Mycoplasma mycoides subsp. mycoides* | N |
| DTA40_07090 | 64% | (*oppF*) oligopeptide ABC transporter, permease component | *Mycoplasma mycoides subsp. mycoides* | N |
| DTA40_07235 | 60% | (*sigA/rpoV*) RNA polymerase sigma factor | *Mycobacterium tuberculosis* | N |
| DTA40_01405 | 64% | (*scpB*) segregation and condensation protein B | *Streptococcus agalactiae* | N |
| DTA40_04480 | 79% | (*cpsY*) transcriptional regulator CpsY | *Streptococcus agalactiae* | N |
| DTA40_00195 | 60% | (*gbpB*) putative peptidoglycan hydrolase | *Streptococcus mutans* | N |
| DTA40_00990 | 71% | (*rgpG*) putative glycosyl transferase N-acetylglucosaminyltransferase), RgpG | *Streptococcus mutans* | N |
| DTA40_02265 | 65% | (*gbpB*) putative peptidoglycan hydrolase | *Streptococcus mutans* | N |
| DTA40_08915 | 88% | (*SMU.322c*) glucose-1-phosphate uridylyltransferase | *Streptococcus mutans* | N |
| DTA40_03310 | 95% | (*eno*) phosphopyruvate hydratase [Streptococcal enolase (CVF153)] | *Streptococcus pneumoniae* | N |
| DTA40_08670 | 95% | (*plr/gapA*) glyceraldehyde-3-phosphate dehydrogenase, type I | *Streptococcus pneumoniae* | N |
| DTA40_03745 | 65% | (*mf2*) MF2 | *Streptococcus pyogenes* | Y |
| DTA40_09915 | 63% | (*htrA/degP*) putative serine protease | *Streptococcus pyogenes* | N |
| DTA40_05380 | 79% | (*SSU98_0573*) Cps2F [Capsule (CVF186)] | *Streptococcus suis* | Y |
| DTA40_00265 | 100% | (*cbpD*) Surface antigen | *Streptococcus thermophilus* | N |
| DTA40_00860 | 100% | (*tig/ropA*) FKBP-type peptidyl-prolyl cis-trans isomerase (trigger factor) | *Streptococcus thermophilus* | N |
| DTA40_02355 | 97% | (*cbpD*) cell wall protein precursor, similar to choline binding protein | *Streptococcus thermophilus* | N |
| DTA40_02360 | 97% | (*cbpD*) cell wall protein precursor, similar to choline binding protein | *Streptococcus thermophilus* | N |
| DTA40_02770 | 99% | (*cshA*) Uncharacterized conserved phage related protein | *Streptococcus thermophilus* | N |
| DTA40_04790 | 100% | (*fbp54*) Fibronectin-binding protein | *Streptococcus thermophilus* | N |
| DTA40_05340 | 97% | (*STER_1057*) Polysaccharide Transporter, PST family | *Streptococcus thermophilus* | N |
| DTA40_05385 | 98% | (*epsE*) exopolysaccharide biosynthesis protein, glycosyl-1-phosphate transferase | *Streptococcus thermophilus* | N |
| DTA40_05395 | 98% | (*STER_1068*) Tyrosine-protein kinase (capsular polysaccharide biosynthesis) | *Streptococcus thermophilus* | N |
| DTA40_05405 | 99% | (*STER_1070*) Capsular polysaccharide biosynthesis protein | *Streptococcus thermophilus* | N |
| DTA40_05410 | 100% | (*STER_1071*) Transcriptional regulator | *Streptococcus thermophilus* | N |
| DTA40_05790 | 97% | (*cshA*) surface-associated protein cshA precursor, truncated | *Streptococcus thermophilus* | Y |
| DTA40_06095 | 100% | (*STER_1222*) dTDP-D-glucose 4,6-dehydratase | *Streptococcus thermophilus* | N |
| DTA40_06100 | 100% | (*STER_1223*) dTDP-4-dehydrorhamnose 3,5-epimerase or related enzyme | *Streptococcus thermophilus* | N |
| DTA40_06105 | 100% | (*rmlA*) glucose-1-phosphate thymidyl transferase | *Streptococcus thermophilus* | N |
| DTA40_06270 | 97% | (*srtA*) Sortase (surface protein transpeptidase) | *Streptococcus thermophilus* | N |
| DTA40_07165 | 99% | (*rgpF*) polysaccharide biosynthesis protein | *Streptococcus thermophilus* | N |
| DTA40_07170 | 93% | (*rgpE*) polysaccharide biosynthesis protein/putative glycosyltransferase | *Streptococcus thermophilus* | N |
| DTA40_07175 | 100% | (*rgpD*) polysaccharide ABC exporter ATP-binding protein | *Streptococcus thermophilus* | N |
| DTA40_07180 | 100% | (*rgpC*) polysaccharide ABC exporter membrane-spanning protein | *Streptococcus thermophilus* | N |
| DTA40_07185 | 99% | (*rgpB*) polysaccharide biosynthesis protein/putative rhamnosyl transferase | *Streptococcus thermophilus* | N |
| DTA40_07190 | 99% | (*rgpA*) polysaccharide biosynthesis protein/putative rhamnosyl transferase | *Streptococcus thermophilus* | N |
| DTA40_07200 | 63% | (*rgpX2*) polysaccharide biosynthesis protein, putative rhamnosyl transferase | *Streptococcus thermophilus* | N |
| DTA40_07205 | 94% | (*rgpX3*) polysaccharide biosynthesis protein, putative transporter | *Streptococcus thermophilus* | N |
| DTA40_07220 | 100% | (*rmlD*) dTDP-4-keto-L-rhamnose reductase | *Streptococcus thermophilus* | N |

**Prediction of virulence-related genes (VRGs) in CS20**

| Protein | Identity | Annotation | Similarity | Pseudogene |
| --- | --- | --- | --- | --- |
| DTA54_01015 | 71% | (*groEL*) chaperonin GroEL | *Clostridium thermocellum* | N |
| DTA54_00650 | 64% | (*CT396*) molecular chaperone DnaK | *Chlamydia trachomatis* | N |
| DTA54_05390 | 63% | (*cpsI*) UDP-galactopyranose mutase | *Enterococcus faecalis* | N |
| DTA54_00980 | 63% | (*uppS*) undecaprenyl diphosphate synthase | *Enterococcus faecium* | N |
| DTA54_01375 | 63% | (*ureA*) urease alpha subunit | *Helicobacter acinonychis* | N |
| DTA54_01395 | 64% | (*ureG*) urease/hydrogenase-associated predicted GTPase UreG | *Helicobacter hepaticus* | N |
| DTA54_05725 | 61% | (*galE*) UDP-glucose 4-epimerase | *Haemophilus somnus* | N |
| DTA54_09445 | 75% | (*lap*) bifunctional acetaldehyde-CoA/alcohol dehydrogenase | *Listeria adhesion* | N |
| DTA54_09450 | 69% | (*lap*) bifunctional aldehyde-alcohol dehydrogenase | *Listeria adhesion* | N |
| DTA54_09460 | 71% | (*lap*) bifunctional aldehyde-alcohol dehydrogenase | *Listeria adhesion* | N |
| DTA54_09465 | 66% | (*lap*) putative alcohol-acetaldehyde dehydrogenase | *Listeria adhesion* | N |
| DTA54_01525 | 61% | (*lisR*) two-component response regulator | *Listeria monocytogenes* | N |
| DTA54_01715 | 64% | (*clpP*) ATP-dependent Clp protease proteolytic subunit | *Listeria monocytogenes* | N |
| DTA54_02320 | 73% | (*tuf*) translation elongation factor Tu | *Mycoplasma mycoides subsp. mycoides* | N |
| DTA54_07385 | 70% | (*oppF*) oligopeptide ABC transporter, permease component | *Mycoplasma mycoides subsp. mycoides* | Y |
| DTA54_07540 | 60% | (*sigA/rpoV*) RNA polymerase sigma factor | *Mycoplasma mycoides subsp. mycoides* | N |
| DTA54_01285 | 64% | (*scpB*) segregation and condensation protein B | *Streptococcus agalactiae* | N |
| DTA54_04565 | 79% | (*cpsY*) transcriptional regulator CpsY | *Streptococcus agalactiae* | N |
| DTA54_04730 | 61% | (*cylG*) 3-ketoacyl-ACP-reductase CylG | *Streptococcus agalactiae* | N |
| DTA54_00195 | 60% | (*gbpB*) putative peptidoglycan hydrolase | *Streptococcus mutans* | N |
| DTA54_02095 | 67% | (*gbpB*) putative peptidoglycan hydrolase | *Streptococcus mutans* | Y |
| DTA54_09205 | 88% | (*SMU.322c*) glucose-1-phosphate uridylyltransferase | *Streptococcus mutans* | N |
| DTA54_00860 | 71% | *(rgpG) putative glycosyl transferase N-acetylglucosaminyltransferase), RgpG* | *Streptococcus mutans* | N |
| DTA54_03130 | 95% | (*eno*) phosphopyruvate hydratase | *Streptococcus pneumoniae* | N |
| DTA54_08970 | 95% | (*plr/gapA*) glyceraldehyde-3-phosphate dehydrogenase, type I | *Streptococcus pneumoniae* | N |
| DTA54_03510 | 62% | (*mf2*) deoxyribonuclease, phage associated | *Streptococcus pyogenes* | N |
| DTA54_03825 | 65% | (*mf2*) deoxyribonuclease, phage associated | *Streptococcus pyogenes* | Y |
| DTA54_10210 | 62% | (*htrA/degP*) putative serine protease | *Streptococcus pyogenes* | N |
| DTA54_07500 | 72% | (*SSA_1515*) hypothetical protein | *Streptococcus sanguinis* | N |
| DTA54_05445 | 79% | (*SSU98_0573*) Cps2F | *Streptococcus suis* | Y |
| DTA54_00265 | 100% | (*cbpD*) Surface antigen | *Streptococcus thermophilus* | N |
| DTA54_00730 | 100% | (*tig/ropA*) FKBP-type peptidyl-prolyl cis-trans isomerase (trigger factor) | *Streptococcus thermophilus* | N |
| DTA54_02185 | 97% | (*cbpD*) cell wall protein precursor, similar to choline binding protein | *Streptococcus thermophilus* | N |
| DTA54_02190 | 97% | (*cbpD*) cell wall protein precursor, similar to choline binding protein | *Streptococcus thermophilus* | N |
| DTA54_02595 | 99% | (*cshA*) Uncharacterized conserved phage related protein | *Streptococcus thermophilus* | N |
| DTA54_04875 | 100% | (*fbp54*) Fibronectin-binding protein | *Streptococcus thermophilus* | N |
| DTA54_05400 | 97% | (*STER_1057*) Polysaccharide Transporter, PST family | *Streptococcus thermophilus* | N |
| DTA54_05450 | 98% | (*epsE*) exopolysaccharide biosynthesis protein, glycosyl-1-phosphate transferase | *Streptococcus thermophilus* | N |
| DTA54_05455 | 99% | (*STER_1068*) Tyrosine-protein kinase (capsular polysaccharide biosynthesis) | *Streptococcus thermophilus* | N |
| DTA54_05460 | 97% | (*epsC*) exopolysaccharide exporter accessory protein | *Streptococcus thermophilus* | N |
| DTA54_05465 | 99% | (*STER_1070*) Capsular polysaccharide biosynthesis protein | *Streptococcus thermophilus* | N |
| DTA54_05470 | 99% | (*STER_1071*) Transcriptional regulator | *Streptococcus thermophilus* | N |
| DTA54_05805 | 83% | (*eps3*) exopolysaccharide biosynthesis protein, glycosyltransferase | *Streptococcus thermophilus* | N |
| DTA54_05810 | 100% | (*eps2*) exopolysaccharide biosynthesis protein, glycosyltransferase | *Streptococcus thermophilus* | N |
| DTA54_05815 | 97% | (*stu1106*) exopolysaccharide biosynthesis protein, truncated | *Streptococcus thermophilus* | N |
| DTA54_05820 | 100% | (*stu1107*) exopolysaccharide biosynthesis protein, truncated | *Streptococcus thermophilus* | Y |
| DTA54_05825 | 97% | (*STER_1067*) Sugar transferase involved in lipopolysaccharide synthesis | *Streptococcus thermophilus* | N |
| DTA54_05830 | 97% | (*STER_1068*) Tyrosine-protein kinase (capsular polysaccharide biosynthesis) | *Streptococcus thermophilus* | N |
| DTA54_05950 | 97% | (*cshA*) surface-associated protein cshA precursor, truncated | *Streptococcus thermophilus* | Y |
| DTA54_06250 | 100% | (*STER_1222*) dTDP-D-glucose 4,6-dehydratase | *Streptococcus thermophilus* | N |
| DTA54_06255 | 100% | (*STER_1223*) dTDP-4-dehydrorhamnose 3,5-epimerase or related enzyme | *Streptococcus thermophilus* | N |
| DTA54_06260 | 100% | (*STER_1224*) dTDP-glucose pyrophosphorylase | *Streptococcus thermophilus* | N |
| DTA54_06425 | 100% | (*srtA*) Sortase (surface protein transpeptidase) | *Streptococcus thermophilus* | N |
| DTA54_06780 | 99% | (*rgpF*) polysaccharide biosynthesis protein | *Streptococcus thermophilus* | N |
| DTA54_06785 | 93% | (*rgpE*) polysaccharide biosynthesis protein/putative glycosyltransferase | *Streptococcus thermophilus* | N |
| DTA54_06790 | 100% | (*rgpD*) polysaccharide ABC exporter ATP-binding protein | *Streptococcus thermophilus* | N |
| DTA54_06795 | 100% | (*rgpC*) polysaccharide ABC exporter membrane-spanning protein | *Streptococcus thermophilus* | N |
| DTA54_06800 | 99% | (*rgpB*) polysaccharide biosynthesis protein/putative rhamnosyl transferase | *Streptococcus thermophilus* | N |
| DTA54_06805 | 99% | (*rgpA*) polysaccharide biosynthesis protein/putative rhamnosyl transferase | *Streptococcus thermophilus* | N |
| DTA54_06815 | 63% | (*rgpX2*) polysaccharide biosynthesis protein, putative rhamnosyl transferase | *Streptococcus thermophilus* | N |
| DTA54_06820 | 94% | (*rgpX3*) polysaccharide biosynthesis protein, putative transporter | *Streptococcus thermophilus* | N |
| DTA54_06835 | 100% | (*rmlD*) dTDP-4-keto-L-rhamnose reductase | *Streptococcus thermophilus* | N |
| DTA54_07465 | 100% | (*STER_1432*) Lipopolysaccharide biosynthesis protein | *Streptococcus thermophilus* | N |
| DTA54_07470 | 100% | (*STER_1433*) Glycosyltransferase | *Streptococcus thermophilus* | N |
| DTA54_07475 | 100% | (*STER_1434*) ABC-type polysaccharide/polyol phosphate transport system, ATPase component | *Streptococcus thermophilus* | N |
| DTA54_07480 | 100% | (*STER_1435*) ABC-type polysaccharide/polyol phosphate export system, permease component | *Streptococcus thermophilus* | N |
| DTA54_07485 | 100% | (*STER_1436*) Glycosyltransferase involved in cell wall biogenesis | *Streptococcus thermophilus* | N |
| DTA54_07490 | 100% | (*STER_1437*) Glycosyltransferase | *Streptococcus thermophilus* | N |
| DTA54_07495 | 100% | (*STER_1438*) Glycosyltransferase involved in cell wall biogenesis | *Streptococcus thermophilus* | N |
| DTA54_07505 | 100% | (*STER_1440*) Glycosyltransferase involved in cell wall biogenesis | *Streptococcus thermophilus* | N |
| DTA54_07510 | 100% | (*STER_1441*) Glycosyltransferase involved in cell wall biogenesis | *Streptococcus thermophilus* | N |
| DTA54_07515 | 100% | (*STER_1442*) Glycosyltransferase involved in cell wall biogenesis | *Streptococcus thermophilus* | N |
| DTA54_07520 | 100% | (*STER_1443*) Membrane protein involved in the export of polysaccharides | *Streptococcus thermophilus* | N |
| DTA54_07525 | 100% | (*STER_1444*) dTDP-4-dehydrorhamnose reductase | *Streptococcus thermophilus* | N |

**Supplementary Table S5 Number of transposases in the four *S. thermophilus* strains**

| Strain | CS5 | CS9 | CS18 | CS20 |
| --- | --- | --- | --- | --- |
| Locus | C1A39_00600  C1A39_00660  C1A39_02615  C1A39_02680  C1A39_02750  C1A39_03595  C1A39_03625  C1A39_04145  C1A39_04150  C1A39_04355  C1A39_04665  C1A39_05970  C1A39_06200  C1A39_06255  C1A39_06790  C1A39_07080  C1A39_08170  C1A39_08785  C1A39_09210  C1A39_09260  C1A39_09300  C1A39_09500  C1A39_09710 | DR994_00635  DR994_00825  DR994_00920  DR994_01705  DR994_02740  DR994_03405  DR994_03500  DR994_03600  DR994_04230  DR994_04375  DR994_08210  DR994_08340  DR994_08790  DR994_09240  DR994_09450 | DTA40_00600  DTA40_00660  DTA40_02615  DTA40_02680  DTA40_02750  DTA40_03755  DTA40_03595  DTA40_03620  DTA40_04145  DTA40_04150  DTA40_04185  DTA40_04355  DTA40_04665  DTA40_05970  DTA40_06200  DTA40_06250  DTA40_06785  DTA40_07075  DTA40_08160  DTA40_08805  DTA40_09215  DTA40_09265  DTA40_09305  DTA40_09510  DTA40_09725 | DTA54_00605  DTA54_01125  DTA54_02445  DTA54_02510  DTA54_02575  DTA54_03555  DTA54_04080  DTA54_04235  DTA54_04240  DTA54_04275  DTA54_04455  DTA54_05475  DTA54_05965  DTA54_06405  DTA54_07115  DTA54_07370  DTA54_09510  DTA54_09570  DTA54_09610 |
| Number | 23 | 15 | 25 | 19 |
| Total(including pseudogenes ) | 132 | 95 | 139 | 116 |

**Supplementary Table S6 The analysis of specific genes in the *eps* cluster of CS9**

| Locus | Annotation | Pseudogene | On the GIs | GC % | Similarity | GC % |
| --- | --- | --- | --- | --- | --- | --- |
| DR994_01880 | Galactofuranosyltransferase | N | N | 39.0 | *Streptococcus gallolyticus* | 40.0 |
| DR994_01885 | Capsular biosynthesis protein | N | N | 35.0 | *Streptococcus salivarius* | 40.0 |
| DR994_01910 | IS256 family transposase | Y | Partial | 40.0 | *Streptococcus macedonicus* | 38.0 |
| DR994_01915 | hypothetical protein | N | Y | 22.0 | *Bacillus* sp. | 38.0 |
| DR994_01920 | hypothetical protein | N | Y | 23.0 | *Lactobacillus delbrueckii* | 50.0 |
| DR994_01925 | glycosyl transferase | N | Y | 24.0 | *Clostridium butyricum* | 29.0 |
| DR994_01930 | glycosyl transferase | N | Y | 21.0 | *Eubacteriaceae bacterium* | 42.0 |
| DR994_01935 | glycosyl transferase | N | Y | 28.0 | *Streptococcus equinus* | 37.0 |
| DR994_01940 | hypothetical protein | N | Y | 37.0 | *Lactococcus lactis* | 35.0 |
| DR994_01945 | hypothetical protein | Y | Partial | 33.0 | *Lactococcus lactis* | 35.0 |
| DR994_01950 | galactose-1-phosphate transferase | N | Y | 30.0 | *Lactococcus lactis* subsp. *cremoris* | 36.0 |
| DR994_01955 | glycosyl transferase | N | Y | 37.0 | *Lactococcus lactis* | 35.0 |

**GIs, Genomic Islands.**

**Supplementary** **Table S7** The growth curve of the four *S. thermophilus* strains in M17*

| Time (h) | CS5 | CS9 | CS18 | CS20 |
| --- | --- | --- | --- | --- |
| 0 | 6.477 ± 0.000 ^a^ | 6.477 ± 0.000 ^a^ | 6.477 ± 0.000 ^a^ | 6.477 ± 0.000 ^a^ |
| 1 | 6.864 ± 0.033 ^b^ | 6.752 ± 0.046 ^a^ | 6.969 ± 0.026 ^c^ | 6.881 ± 0.063 ^b^ |
| 2 | 7.575 ± 0.041 ^c^ | 6.920 ± 0.030 ^a^ | 7.806 ± 0.018 ^d^ | 7.174 ± 0.049 ^b^ |
| 3 | 8.226 ± 0.004 ^c^ | 7.417 ± 0.067 ^a^ | 8.230 ± 0.008 ^c^ | 7.799 ± 0.018 ^b^ |
| 4 | 8.281 ± 0.002 ^c^ | 7.885 ± 0.009 ^a^ | 8.284 ± 0.015 ^c^ | 8.142 ± 0.006 ^b^ |
| 5 | 8.297 ± 0.002 ^c^ | 8.036 ± 0.008 ^a^ | 8.283 ± 0.002 ^c^ | 8.201 ± 0.007 ^b^ |
| 6 | 8.308 ± 0.004 ^c^ | 8.083 ± 0.009 ^a^ | 8.300 ± 0.003 ^c^ | 8.224 ± 0.004 ^b^ |
| 7 | 8.320 ± 0.004 ^d^ | 8.111 ± 0.007 ^a^ | 8.303 ± 0.004 ^c^ | 8.235 ± 0.001 ^b^ |
| 8 | 8.328 ± 0.002 ^d^ | 8.121 ± 0.003 ^a^ | 8.312 ± 0.002 ^c^ | 8.224 ± 0.004 ^b^ |
| 10 | 8.326 ± 0.004 ^d^ | 8.118 ± 0.005 ^a^ | 8.310 ± 0.002 ^c^ | 8.235 ± 0.001 ^b^ |
| 12 | 8.324 ± 0.006 ^d^ | 8.115 ± 0.002 ^a^ | 8.306 ± 0.001 ^c^ | 8.245 ± 0.004 ^b^ |
| 14 | 8.324 ± 0.002 ^d^ | 8.111 ± 0.003 ^a^ | 8.307 ± 0.002 ^c^ | 8.246 ± 0.001 ^b^ |
| 16 | 8.326 ± 0.002 ^d^ | 8.113 ± 0.002 ^a^ | 8.305 ± 0.002 ^c^ | 8.246 ± 0.004 ^b^ |
| 18 | 8.320 ± 0.004 ^d^ | 8.108 ± 0.002 ^a^ | 8.302 ± 0.001 ^c^ | 8.243 ± 0.002 ^b^ |
| 20 | 8.318 ± 0.002 ^d^ | 8.108 ± 0.002 ^a^ | 8.301 ± 0.004 ^c^ | 8.237 ± 0.004 ^b^ |
| 22 | 8.315 ± 0.001 ^d^ | 8.104 ± 0.003 ^a^ | 8.296 ± 0.003 ^c^ | 8.235 ± 0.004 ^b^ |
| 24 | 8.314 ± 0.002 ^d^ | 8.102 ± 0.002 ^a^ | 8.295 ± 0.003 ^c^ | 8.233 ± 0.003 ^b^ |

*, The growth ability was expressed as lgCFU, each value represents the mean of three measurements ±SD. The homogeneous subsets were defined by means of Duncan using SPSS software. a, b, c, d represent different significant differences respectively; Subset for α=0.05.

**Supplementary** **Table S8** The specific growth rates and final biomasses of strains of CS5, CS9, CS18, and CS20 in M17*.

| Time (h) | CS5 | CS9 | CS18 | CS20 |
| --- | --- | --- | --- | --- |
| 1 | 0.397± 0.033^b^ | 0.275 ± 0.045^a^ | 0.492 ± 0.026 ^c^ | 0.404 ± 0.063^d^ |
| 2 | 0.710 ± 0.073 ^c^ | 0.168 ± 0.004 ^a^ | 0.836 ± 0.040 ^d^ | 0.293 ± 0.014 ^b^ |
| 3 | 0.651 ± 0.045 ^d^ | 0.497 ± 0.039 ^b^ | 0.424 ± 0.016 ^a^ | 0.625 ± 0.003 ^c^ |
| 4 | 0.055 ± 0.003 ^a^ | 0.468 ± 0.006 ^c^ | 0.054 ± 0.012 ^a^ | 0.342 ± 0.022 ^b^ |
| 5 | 0.015 ± 0.004 ^a^ | 0.151 ± 0.008 ^c^ | -0.001 ± 0.013 ^a^ | 0.059 ± 0.001 ^b^ |
| 6 | 0.012 ± 0.004 ^a^ | 0.046 ± 0.015 ^b^ | 0.016 ± 0.005 ^a^ | 0.022 ± 0.006 ^a^ |
| 7 | 0.012 ± 0.004 ^a b^ | 0.028± 0.010 ^b^ | 0.004 ± 0.004 ^a^ | 0.011± 0.003 ^a b^ |
| 8 | 0.008 ± 0.002 ^a^ | 0.01± 0.006 ^a^ | 0.001± 0.004 ^a^ | 0.011 ± 0.002 ^a^ |
| 10 | -0.001 ± 0.001 ^a^ | -0.002 ± 0.002 ^a^ | -0.002 ± 0.002 ^a^ | 0.002 ± 0.004 ^a^ |
| 12 | -0.002 ± 0.005 ^a^ | -0.003± 0.003 ^a^ | -0.03± 0.001 ^a^ | 0.000 ± 0.004 ^a^ |
| 24 | -0.004 ± 0.006 ^a^ | -0.006 ± 0.003 ^a^ | 0.000 ± 0.006 ^a^ | 0.004 ± 0.006 ^a^ |
| 24h lgCFU^#^ | 8.314 ± 0.002 ^d^ | 8.102 ± 0.002 ^a^ | 8.295 ± 0.003 ^c^ | 8.233 ± 0.003 ^b^ |

^*^, Specific growth rate was expressed as lg CFU_1_ /CFU_0_ (where CFU_0_ is the number of viable cells before and CFU_1_ is the number of viable cells after). Each value represents the mean of three measurements ±SD. The homogeneous subsets were defined by means of Ducan using SPSS software. a, b, c, d represent different significant differences respectively; Subset for α=0.05.

^#^, The final biomass (growth yield = final lgCFU).

**Supplementary** **Table S9** The growth curve of the four *S. thermophilus* strains in Milk.

| Time (h) | CS5 | CS9 | CS18 | CS20 |
| --- | --- | --- | --- | --- |
| 0 | 6.398 ± 0.000 ^a^ | 6.398 ± 0.000 ^a^ | 6.398 ± 0.000 ^a^ | 6.398 ± 0.000 ^a^ |
| 1 | 6.699 ± 0.000 ^a^ | 6.599 ± 0.174 ^a^ | 6.799 ± 0.013 ^b^ | 6.758 ± 0.102 ^a^ |
| 2 | 7.333 ± 0.056 ^b^ | 7.198 ± 0.039 ^a^ | 7.538 ± 0.140 ^c^ | 7.257 ± 0.089 ^b^ |
| 3 | 7.539 ± 0.070 ^b^ | 7.262 ± 0.033 ^a^ | 7.668 ± 0.036 ^c^ | 7.541 ± 0.063 ^b c^ |
| 4 | 7.771 ± 0.028 ^b c^ | 7.392 ± 0.088 ^a^ | 7.950 ± 0.079 ^c^ | 7.832 ± 0.102 ^b^ |
| 5 | 7.912 ± 0.020 ^c^ | 7.477 ± 0.001 ^a^ | 8.079 ± 0.024 ^d^ | 7.850 ± 0.009 ^b^ |
| 6 | 8.024 ± 0.016 ^c^ | 7.636± 0.038 ^a^ | 8.130 ± 0.016 ^d^ | 7.954 ± 0.021 ^b^ |
| 7 | 8.067 ± 0.022 ^c^ | 7.790 ± 0.020 ^a^ | 8.145 ± 0.035 ^c^ | 8.014 ± 0.006 ^b^ |
| 8 | 8.117 ± 0.013 ^c^ | 7.911± 0.034 ^a^ | 8.230± 0.006 ^c^ | 8.044 ± 0.034 ^b^ |
| 10 | 8.226 ± 0.010 ^c^ | 7.981 ± 0.035 ^a^ | 8.253 ± 0.009 ^d^ | 8.149 ± 0.012 ^b^ |
| 12 | 8.217 ± 0.007 ^c^ | 8.097 ± 0.015 ^a^ | 8.251 ± 0.004 ^c^ | 8.164 ± 0.011 ^b^ |
| 14 | 8.235 ± 0.013 ^c^ | 8.136 ± 0.012 ^a^ | 8.249 ± 0.006 ^d^ | 8.166 ± 0.010 ^b^ |
| 16 | 8.222 ± 0.007 ^c^ | 8.136 ± 0.032 ^a^ | 8.248 ± 0.011 ^d^ | 8.171 ± 0.011 ^b^ |
| 18 | 8.215 ± 0.010 ^c^ | 8.144 ± 0.008 ^a^ | 8.249 ± 0.011 ^d^ | 8.161 ± 0.026 ^b^ |
| 20 | 8.186 ± 0.011 ^c^ | 8.103 ± 0.013 ^a^ | 8.255 ± 0.006 ^d^ | 8.149 ± 0.012 ^b^ |
| 22 | 8.183 ± 0.007 ^c^ | 8.091 ± 0.019 ^a^ | 8.248 ± 0.011 ^d^ | 8.143 ± 0.016 ^b^ |
| 24 | 8.176 ± 0.007 ^c^ | 8.076 ± 0.005 ^a^ | 8.248 ± 0.009 ^d^ | - 1. ± 0.017 ^b^ |

**Supplementary Table S10** The specific growth rates and final biomasses of strains of CS5, CS9, CS18, and CS20 in Milk*.

| Time (h) | CS5 | CS9 | CS18 | CS20 |
| --- | --- | --- | --- | --- |
| 1 | 0.301± 0.000 ^b^ | 0.201 ± 0.012 ^a^ | 0.401 ± 0.014 ^d^ | 0.360 ± 0.056 ^c^ |
| 2 | 0.634 ± 0.028 ^c^ | 0.600 ± 0.067 ^b^ | 0.8739 ± 0.017 ^d^ | 0.499 ± 0.065 ^a^ |
| 3 | 0.206 ± 0.012 ^c^ | 0.064 ± 0.025 ^a^ | 0.130 ± 0.052 ^b^ | 0.284 ± 0.013 ^d^ |
| 4 | 0.232 ± 0.025 ^b^ | 0.130± 0.027 ^a^ | 0.282± 0.021 ^c^ | 0.291 ± 0.020 ^c^ |
| 5 | 0.140 ± 0.004 ^c^ | 0.085 ± 0.004 ^b^ | 0.004 ± 0.001 ^a^ | 0.018 ± 0.002 ^a^ |
| 6 | 0.113 ± 0.003 ^a b^ | 0.159 ± 0.004 ^c^ | 0.125 ± 0.028 ^b^ | 0.103 ± 0.006 ^a^ |
| 7 | 0.042 ± 0.003 ^a^ | 0.154 ± 0.009 ^B^ | 0.051 ± 0.004 ^a^ | 0.060 ± 0.007 ^a^ |
| 8 | 0.050 ± 0.004 ^b^ | 0.121± 0.007 ^c^ | 0.015 ± 0.004 ^a^ | 0.030± 0.003 ^a^ |
| 10 | 0.11 ± 0.001 ^b^ | 0.069 ± 0.002 ^a^ | 0.085 ± 0.010 ^a^ | 0.105 ± 0.012 ^b^ |
| 12 | -0.009 ± 0.001 ^a^ | 0.116± 0.010 ^c^ | 0.023 ± 0.002 ^b^ | 0.015± 0.001 ^b^ |
| 24 | -0.041 ± 0.001 ^a^ | -0.021 ± 0.005 ^b^ | -0.004 ± 0.001 ^c^ | -0.031 ± 0.002 ^a b^ |
| 24h lgCFU^#^ | 8.176 ± 0.007 ^c^ | 8.076 ± 0.005 ^a^ | 8.249 ± 0.010 ^d^ | 8.133 ± 0.017 ^b^ |

^*^, Specific growth rate was expressed as lg CFU_1_ /CFU_0_ (where CFU_0_ is the number of viable cells before and CFU_1_ is the number of viable cells after). Each value represents the mean of three measurements ±SD. The homogeneous subsets were defined by means of Ducan using SPSS software. a, b, c, d represent different significant differences respectively; Subset for α=0.05.

^#^, The final biomass (growth yield = final lgCFU).

**Supplementary Table S11** Resistance ability of the four *S. thermophilus* strains

| Strain | Different treatment (LgCFU) | | | Survival Rate (%) | |
| --- | --- | --- | --- | --- | --- |
|  | Acid | Bile | Normal | Acid | Bile |
| CS5 | 7.45±0.04 | 7.83±0.03 | 8.06±0.04 | 24.67 | 53.17 |
| CS9 | 7.89±0.02 | 7.91±0.02 | 7.99±0.02 | 79.08 | 75.00 |
| CS18 | 7.93±0.08 | 8.02±0.07 | 8.05±0.07 | 75.81 | 94.40 |
| CS20 | 7.70±0.07 | 7.94±0.03 | 8.04±0.04 | 47.03 | 79.91 |

*The survival cells under different treatment were expressed as lgCFU. Each value represents the mean of three measurements ±SD.
